# McMYB12 Transcription Factors Co-regulate Proanthocyanidin and Anthocyanin Biosynthesis in *Malus* Crabapple

**DOI:** 10.1038/srep43715

**Published:** 2017-03-03

**Authors:** Ji Tian, Jie Zhang, Zhen-yun Han, Ting-ting Song, Jin-yan Li, Ya-ru Wang, Yun-cong Yao

**Affiliations:** 1 Department of Plant Science and Technology, Beijing University of Agriculture, Beijing, China; 2 Key Laboratory of New Technology in Agricultural Application of Beijing, Beijing University of Agriculture, Beijing, China; 3 Beijing Collaborative innovation center or eco-environmental improvement with forestry and fruit trees, Beijing, China.

## Abstract

The flavonoid compounds, proanthocyanidins (PAs), protect plants from biotic stresses, contribute to the taste of many fruits, and are beneficial to human health in the form of dietary antioxidants. In this study, we functionally characterized two *Malus* crabapple R2R3-MYB transcription factors, McMYB12a and McMYB12b, which co-regulate PAs and anthocyanin biosynthesis. McMYB12a was shown to be mainly responsible for upregulating the expression of anthocyanin biosynthetic genes by binding to their promoters, but to be only partially responsible for regulating PAs biosynthetic genes. In contrast, McMYB12b showed preferential binding to the promoters of PAs biosynthetic genes. Overexpression of *McMYB12a* and *McMYB12b* in tobacco (*Nicotiana tabacum*) altered the expression of flavonoid biosynthetic genes and promoted the accumulation of PAs and anthocyanins in tobacco petals. Conversely, transient silencing their expression in crabapple plants, using a conserved gene region, resulted in reduced PAs and anthocyanin production a green leaf phenotype. Meanwhile, transient overexpression of the two genes and silenced *McMYB12s* in apple (*Malus domestica*) fruit had a similar effect as overexpression in tobacco and silenced in crabapple. This study reveals a new mechanism for the coordinated regulation of PAs and anthocyanin accumulation in crabapple leaves, which depends on an auto-regulatory balance involving *McMYB12a* and *McMYB12b* expression.

Various classes of metabolites are produced via the flavonoid pathway, including flavonols, anthocyanins, and proanthocyanidins (PAs), which are widely distributed throughout the plant kingdom^1^. Flavonols provide protection against UV radiation^2^, contribute to pollen fertility^3^, and act as co-pigments, together with anthocyanins, in organ coloration^4^. Anthocyanins are secondary metabolites that impart characteristic red, blue, and purple colors to plant tissues and, as such, play an important role in plant reproduction, by attracting pollinators and seed dispersers, as well as protecting plants from photo-oxidative stress^1^. PAs, which are also called condensed tannins, and are present in the seed coats of many species, confer astringency to fruits, leaves, stems, and seeds, thereby functioning as herbivore feeding deterrents^5^,^6^,^7^. Dietary PAs and anthocyanins are present in many fruits and plant products, such as wine, fruit juices, and teas, and are valued for both their taste and health benefits. For example, they are recognized as having pharmaceutical properties that can help prevent heart disease, cancer, diabetes and degenerative conditions, such as Alzheimer’s disease^8^,^9^.

Members of each class of flavonoid (flavonols, anthocyanins and proanthocyanidins) are synthesized via a multi-step enzymatic reaction branching from the common core flavonoid pathway (Fig. 1). To summarize, the colorless flavonols are synthesized from dihydroflavonols by flavonol synthase (FLS) enzymes^10^. The biosynthetic route to anthocyanins is catalyzed by the cooperative action of dihydroflavonol 4-reductase (DFR), anthocyanidin synthase (ANS) and uridine diphosphate (UDP)-glucose: flavonoid-*O*-glycosyltransferase (UFGT)^11^. Leucoanthocyanidin reductase (LAR) and anthocyanidin reductase (ANR) act at the branch of the flavonoid pathway leading to the synthesis of PAs, such as catechin and epicatechin^12^,^13^,^14^.

Apple fruits vary considerably in color, ranging from yellow, green, or red, along with varied differences in red color pigmentation patterns. One of the most common anthocyanin a pigment is cyanidin, which, in the form of cyanidin 3-*O*-galactoside, is the pigment primarily responsible for red colouration in apple skin^11^. Meanwhile, PAs are abundant in vegetative tissues such as leaves, stems and roots, and are also found in fruit in the hypodermis in apple^15^,^16^. Cyanidin was the main coloration anthocyanin component in *Malus* crabapple leaves^17^. But the PAs accumulation and biosynthesis in crabapple is still unknown.

The flavonoid pathway is thought to be primarily regulated at the level of the transcription of the genes encoding the associated biosynthetic enzymes^1^. Several transcription factors (TFs) that control their transcription have been isolated from a diverse group of plants^18^,^19^,^20^,^21^,^22^. The R2R3-MYB family is one of the largest groups of plant transcriptional regulators, and individual members have been shown to have diverse roles in development, physiology and metabolism^23^,^24^,^25^, including the regulation of anthocyanin biosynthesis. For example, the grapevine (*Vitis vinifera*) R2R3-MYB proteins VvMYBA1 and VvMYBA2 function as regulators of anthocyanin levels^26^,^27^,^28^, while VvMYBPA1, VvMYBPA2 and VvMYBPAR promote PAs accumulation^29^,^30^,^31^, and VvMYBF1 is putatively involved in flavonol synthesis^10^. In addition, VvMYB5a and VvMYB5b have been partially characterized and are thought to regulate structural genes involved in the general flavonoid pathway at different stages of berry development^32^,^33^. Transient expression of these two TFs in tobacco (*Nicotiana tabacum*) resulted in the activation of several flavonoid pathway genes, and when overexpressed, the biosynthesis of anthocyanins, flavonols, tannins and lignins in reproductive organs was increased^32^,^33^. In apple (*Malus domestica*), the R2R3-MYB genes *MdMYB1, MdMYB10*, and *MdMYBA* have been characterized and shown to influence anthocyanin accumulation and fruit coloration^11^,^34^,^35^. *MdMYB1* and *MdMYBA* transcript levels were reported to increase in dark-grown apples following exposure to light^34^,^35^,^36^, while *MdMYB10* was shown to control anthocyanin production in apple fruit and leaves, and to bind directly to its own promoter in an auto-regulatory-loop, leading to substantial anthocyanin accumulation^37^. It was recently reported that increased expression of *MdMYB110a*, a paralog of *MdMYB10,* was associated with a red-flesh cortex phenotype, and when over-expressed in tobacco, resulted in the up-regulation of anthocyanin biosynthesis^38^. *MdMYB3* is involved in transcriptional activation of several flavonoid pathway genes. Moreover, this TF not only regulates the accumulation of anthocyanin in the skin of apple fruits, but it is also involved in the regulation of flower development, particularly that of pistil development^39^.

*MdMYB9* and *MdMYB11* which have been characterized to play roles in the regulation of PA biosynthesis were isolated recently in apple. Promoter binding assay showed that MdMYB9 and MdMYB11 were able to activate the PA-specific biosynthesis genes promoter, and overexpression of *MdMYB9* or *MdMYB11* promoted not only anthocyanin but also PA accumulation in apple. In addition, these two genes are stress and JA responsive^40^,^41^. However, while these studies contribute to the overall understanding of flavonoid regulation, the transcriptional regulation of PAs levels in *Malus* crabapple is still not well understood.

*Malus* crabapple is an important ornamental woody plant, which belongs to the Rosaceae, *Malus Mill* family. *Malus* crabapples were originated in Europe and cultivated almost 240 years all over the world^42^. The genome of crabapple is almost same with the total genome of *Malus domestica* (603.9 Mb in apple)^43^. And *Malus* crabapple represents a useful model system as it has one of the most economically important ornamental apple germplasm resources, due to the high flavonoid levels in its leaves, flowers and fruits^44^. Furthermore, the abundant levels of flavonoids in the leaves and fruits represent an excellent source of antioxidants for use as food nutrition additives^45^, and the consumption of crabapple leaf tea as a health beverage is gaining popularity in Asia. In the current study, we compared PAs biosynthesis in two commercial crabapple cultivars which planted in northern area in China: ‘Royalty’ with ever-red leaves and ‘Flame’ with ever-green leaves (Originated in America and have relative closely genetic background)^42^. We also investigated the transcriptional regulation of PAs biosynthesis by functionally characterizing the *Malus* crabapple TFs, McMYB12a and McMYB12b, which were identified as potential targets because of their similarity to identified PAs regulators in grape.

We present results indicating that both McMYB12 proteins are putative regulators of the PAs and anthocyanins branches of the flavonoid pathway. This reinforces the idea that the biosynthesis of flavonoid compounds is a complex process involving transcriptional network changes, such that TFs serving as main regulators in one branch of the flavonoid pathway may coordinately regulate other flavonoid branches that share an upstream substrate.

## Results

### Expression of McMYB12 TFs correlates with PAs accumulation during crabapple leaf development

We previously reported that the anthocyanin concentration in ever-red leaves, was significantly higher than that in ever-green leaves^17^, and that a competitive relationship between anthocyanin and flavonol is important for leaf coloration^46^. We characterized PAs accumulation patterns during five leaf developmental stages (1–5) of the ever-red leaf cultivar ‘Royalty’ and the ever-green leaf cultivar ‘Flame’, using high pressure liquid chromatography (HPLC) (Fig. 2A). We observed that in the ever-red ‘Royalty’ cultivar, the abundance of anthocyanins (cyanidin-3-*O*-glucoside) and PAs was relatively high in the first three stages, before decreasing to low levels in the last two stages, while epicatechin accumulation gradually increased. In the ever-green leaved ‘Flame’ cultivar, anthocyanins were almost undetectable, whereas relatively high levels of PAs and epicatechin were measured during leaf development (Fig. 2B). The concentration of flavonols (quercetin-3-*O*-rhamnoside and rutin) gradually decreased in ‘Royalty’ and ‘Flame’ leaves during leaf development, with the exception of stage 4 in ‘Flame’. The same trends of decreasing levels were also observed for phlorizin in ‘Flame’ and for in avicularin in ‘Royalty’. In contrast, avicularin abundance increased during the development of ‘Flame’ leaves, while there was no apparent variation in amount of the phlorizin in ‘Royalty’ leaves.

We also evaluated the abundance of these flavonoid compounds in the petals, fruit peel and fruit flesh of ‘Royalty’ and ‘Flame’ (Supplemental Figs 1A, 2A and 3A). In petals, the concentrations of anthocyanins and avicularin were higher in ‘Royalty’ than in ‘Flame’, and decreased in these two cultivars during petals development. The concentration of (−)-epicatechin significantly increased in the last development stage of petals in ‘Royalty’ but the compound was not detected in ‘Flame’ petals. The amounts of quercetin-3-*O*-rhamnoside and phlorizin were similar in the two cultivars (Supplemental Fig. 1B).

The concentrations of most flavonoids decreased in fruit peels during fruit development in ‘Royalty’ and ‘Flame’. Anthocyanin and avicularin were not detected in ‘Flame’ peels and the concentrations of PAs ((−)-epicatechin and procyanidin B2) in ‘Royalty’ were higher than in ‘Flame’ (Supplemental Fig. 2B). We observed a gradual decrease in procyanidin B2 and quercetin-3-*O*-rhamnoside levels during fruit flesh development, while anthocyanins and quercetin-3-*O*-rhamnoside were not detected in ‘Flame’ fruit flesh, and (−)-epicatechin was not detected in ‘Royalty’ fruit flesh (Supplemental Fig. 3B).

Taken together, these results suggested that the red crabapple color is related to anthocyanin accumulation, and that the levels of PAs show substantial variation during leaf, petal and fruit development.

Consistent with this idea, the expression of PAs biosynthetic genes (*McANR1, McANR2, McLAR1* and *McLAR2*) and anthocyanin biosynthetic genes *(McCHS, McF3H, McF3’H, McANS* and *McUFGT*) gradually increased during leaf development, peaking at stage 3 or 4, before gradually decreasing in ‘Royalty’, while they gradually decreased during leaf development in ‘Flame’, as determined by quantitative real-time PCR (qRT-PCR). Expression of the flavonol biosynthesis gene, *McFLS*, gradually decreased in the leaves of ‘Royalty’ and but increased in the leaves of ‘Flame’ during leaf development. This suggested that *McFLS* expression is closely related with flavonol accumulation during the development crabapple leaves (Fig. 2C). The transcript levels of *McANR1, McANR2, McLAR1* and *McLAR2* were also measured during the development of petals, fruit peels and fruit flesh in these two cultivars (Supplemental Figs 1C, 2C and 3C) and were observed to correlate with PA accumulation.

Two full-length *McMYB12s* cDNA were cloned from crabapple cDNA libraries by homologous cloning which are similar to VvMYB5a and VvMYB5b, respectively. We hypothesized that the corresponding genes, which we named *McMYB12a* (KJ020112) and *McMYB12b* (KJ020111), might act as key regulators of PAs biosynthesis. The full-length *McMYB12a* and *McMYB12b* cDNAs were 2,087 and 2,213 bp long, respectively, and their open reading frames were predicted to encode proteins of 364 and 374 amino acids, with 92% sequence identity in the R2R3 domain (Fig. 3A). The two McMYB12 proteins showed sequence homology with known PAs regulatory MYB family proteins from other species^29^,^30^,^32^,^33^, and each was predicted to contain a DNA-binding ID domain motif, a C1 motif and a C3 motif. Phylogenetic analysis showed that McMYB12a and McMYB12b are closely related to the potential PAs regulators MdMYB12, VvMYB5a and VvMYB5b (Fig. 3B). Their expression patterns were found to be positively correlated with the expression of the PAs biosynthetic genes *McANR1, McANR2, McLAR1* and *McLAR2* in crabapple leaves, petals, fruit peel and fruit flesh (Fig. 2C, Supplemental Figs 1C, 2C and 3C). Meanwhile, the correlation analysis showed the expression of *McMYB12a* and *McMYB12b* are closely related the accmulation of (−)-epicatechin and procyanidin B2 in crabapple leaves, petals, fruit peels and fruit flesh (Supplemental Fig. 4). This result supported the hypothesis that the two McMYB12 proteins play a role in the PAs synthesis pathway.

To elucidate the reason underlying this expression difference of *McMYB12a* and *McMYB12b* in two crabapple cultivars, a 1112 bp of *McMYB12a* promoter in ‘Royalty’ (KY51104) and a 1107 bp of *McMYB12a* promoter in ‘Flame’ (KY51105), and a 1191 bp of *McMYB12b* promoter in ‘Royalty’ (KY51103) and a 1191 bp of *McMYB12b* promoter in ‘Flame’ (KY51106) were cloned from crabapple leaves. The results showed that there are no significantly structure difference and GUS activity variation of *McMYB12s* promoters between ‘Royalty’ and ‘Flame’ cultivars (Supplemental Fig. 5).

### McMYB12 proteins activate the promoters of genes required for PAs and anthocyanin synthesis

The two *McMYB12* genes and several flavonoid biosynthetic genes exhibited similar expression patterns during crabapple leaf development. To test the hypothesis that PAs biosynthetic genes might be targets of McMYB12a and/or McMYB12b in crabapple, we evaluated their binding to the promoters of *McCHS, McF3’H, McDFR, McANS, McUFGT, McFLS, McANR1, McANR2, McLAR1* and *McLAR2*, using a yeast one hybrid assay. *LacZ* activity was detected in yeast cells harboring pJG4-5-*McMYB12a* together with pLacZi-*proMcCHS*, pLacZi-*proMcANS*, pLacZi-*proMcANR1*, pLacZi-*proMcANR2* and pLacZi-*proMcLAR2*, but not in yeast harboring pLacZi-*proMcF3’H*, pLacZi-*proMcDFR*, pLacZi-*proMcUFGT*, pLacZi-*proMcFLS* and pLacZi-*proMcLAR1* together with pJG4-5-*McMYB12a*, or in the control. *LacZ* activity was detected in yeast harboring pJG4-5-*McMYB12b* together with pLacZi-*proMcANS*, pLacZi-*proMcANR1*, pLacZi-*proMcANR2*, pLacZi-*proMcLAR1* and pLacZi-*proMcLAR2*, but not in yeast harboring pLacZi-*proMcCHS*, pLacZi-*proMcF3’H*, pLacZi-*proMcDFR*, pLacZi-*proMcUFGT* and pLacZi-*proMcFLS* together with pJG4-5-*McMYB12b*, or in the control. From this we deduced that *McCHS, McANS, McANR1, McANR2, McLAR2* and *McANS, McANR1, McANR2, McLAR1, McLAR2*, which resulted in positive binding results, may be the downstream target genes of *McMYB12a* and *McMYB12b*, respectively (Fig. 4A).

To further determine whether McMYB12 proteins binds directly to the promoter of the flavonoid biosynthetic genes identified in the yeast one hybrid assay, we performed an electrophoretic mobility shift assay (EMSA). Biotin labeled probes were designed according to the CAACTG element in *proMcCHS* and *proMcANS*, the AACCTAA element in *proMcANR1*, the TAACTG element in *proMcANR2* and the TATCC element in *proMcLAR1* and *proMcLAR2* (Table S2). We found that McMYB12a bound to the biotin labeled *proMcCHS, proMcANS, proMcANR1, proMcANR2* and *proMcLAR2* probes, and that McMYB12b bound to the biotin labeled *proMcANS, proMcANR1, proMcANR2, proMcLAR1* and *McLAR2* probes (Fig. 4B). This binding diminished gradually with an increasing concentration of un-labeled probe, while there was no evidence of competition using a mutated probe (Fig. 4B), indicating that *McCHS, McANS, McANR1, McANR2* and *McLAR2* are direct targets of McMYB12a, and that *McANS, McANR1, McANR2, McLAR1* and *McLAR2* are direct targets of *McMYB12b*. Furthermore, slightly *LacZ* activity between pJG4-5-*McMYB12a* and pLacZi-*proLAR1* in yeast cell was proved as false positive using EMSA. These data support the hypothesis that *McMYB12a* and *McMYB12b* are regulators of the PAs biosynthetic pathway. Furthermore, the results suggest that McMYB12a and McMYB12b have different functions by binding directly to different loci in the promoters of the downstream flavonoid biosynthetic genes.

To identify the structural genes of the flavonoid pathway activated by McMYB12s, transient expression experiments using dual luciferase system were conducted. The promoters of flavonoids biosynthesis gene were fused to luciferase as was the previously tested *DFR* promoter from apple^11^. *McMYB12s* were driven by the cauliflower mosaic virus 35 S promoter and were co-transformed with *McbHLH3* (interacted with McMYB12a and McMYB12b; Supplemental Fig. 6) construct. The results showed that the promoters of *McCHS, McANS, McANR1, McANR2* and *McLAR2* were strongly activated by McMYB12a with McbHLH3, and the promoters of *McANS, McANR1, McANR2, McLAR1* and *McLAR2* were strongly activated by McMYB12b with McbHLH3 in tobacco leaves (Fig. 5).

### Altered *McMYB12a* and *McMYB12b* gene expression in tobacco flowers affects the accumulation of anthocyanin-derived and PAs-derived compounds

In order to establish the function of *McMYB12a* and *McMYB12b*, constructs containing each full-length cDNA driven by the constitutive 35 S promoter were transformed separately into tobacco (*Nicotiana tabacum*) plants. The strongest phenotypic effects were seen in the petals of three *McMYB12a* transgenic lines and three *McMYB12b* transgenic lines, where the pigmentation was much greater in *McMYB12a* overexpressing plants than in the wild type controls and *McMYB12b* transgenic lines (Fig. 6A). Transgenic *McMYB12b* petals appeared lighter pink than the control flowers, with a white circle in the middle (Fig. 6A).

HPLC analysis indicated significantly higher levels of anthocyanins and epicatechin in the petals of three *McMYB12a* transgenic lines compared with wild-type plants. The epicatechin and procyanidin contents of the petals in three different *McMYB12b* transgenic lines were at least twice than the amount measured in the petals of the control plants. The HPLC analysis also revealed an increase in the concentration of rutin and quercetin-3-*O*-rhamnoside in the transgenic lines. Finally, the anthocyanin content of the petals in the *McMYB12b* transgenic lines was notably lower than wild type (Fig. 6B).

The tobacco overexpression lines had clearly detectable levels of *McMYB12a* or *McMYB12b* transcripts in their petals (Fig. 6C). Gene expression analysis further revealed that the transcript levels of the putative MYB12a target genes, *NtCHS, NtANS, NtANR1* and *NtANR2*, were significantly higher in the three different *McMYB12a*-overexpressing lines compared with the control lines. Similarly, expression of the putative MYB12b target genes, *NtANS, NtANR1, NtANR2* and *NtLAR*, was significantly up-regulated in the three different *McMYB12b* overexpression lines (Fig. 6C). The expression levels of the other downstream flavonoid biosynthesis genes, *NtF3’H, NtUFGT* and *NtFLS*, were also significantly increased (Fig. 6C). Furthermore, we also tested the expression of endogenous tobacco flavonoids regulation factors, the results showed the the expression level of *NtAn1a, NtAn1b* and *NtAn2* increased in *McMYB12s* overexpressed tobacco in pBI121-*McMYB12a* 5# and pBI121-*McMYB12b* 9#, but not in other trangenic lines. So we suppose that there’s no obvious expression correlation between *McMYB12s* and *NtAns* (Supplemental Fig. 8A).

In addition, dimethylaminocinnamaldehyde (DMACA) staining revealed that the accumulation of PAs in the transgenic seeds was higher than in control seeds, while HPLC confirmed that the concentration of PAs was at least 2 fold higher in the *McMYB12a* and *McMYB12b* transgenic lines than that in control seeds (Supplemental Fig. 7A,B). And there is no obviously variation of flavonoids content in McMYB12s transgenic leaves compared with control leaves (Supplemental Fig. 7C,D).

Overexpression *McMYB12s* has the same phenotypes with overexpression *VvMYB5a* and *VvMYB5b* in tobacco. The flavonoids biosynthesis pathway was only activated in reproductive organs, not in vegetative organs. We speculated that the tobacco leaves maybe lack in the flavonoids biosynthesis substrates. These results also indicate that *McMYB12a* and *McMYB12b* can regulate flavonoid metabolism in tobacco flowers and seeds; however, the regulatory modes and function of these two TFs may differ depending on the target genes.

### Overexpression or silencing of *McMYB12a* and *McMYB12b* expression in crabapple alters the accumulation of anthocyanin- and PAs-derived compounds

To further elucidate the role of the *McMYB12* genes, we suppressed their expression in the leaves of the red-leaved crabapple cultivar ‘Royalty’ by virus-induced gene silencing (VIGS), using the TRV-GFP vector. This contains a conserved McMYB12 region, and so in principal can silence the expression of both *McMYB12a* and *McMYB12b*. Crabapple tissue culture plantlets infiltrated with the virus containing the TRV-GFP-*McMYB12s* construct developed a faded-purple phenotype in the new buds at 14 days post-infection (dpi). In contrast, the plantlets infiltrated with the empty vector, pTRV2-GFP, rapidly accumulated anthocyanins, resulting in red coloration in the upper young leaves (Fig. 7A). In addition, GFP fluorescence was observed in leaf veins and new buds at 14 dpi, indicating that the virus spread through the plants. HPLC analyses confirmed that the anthocyanin concentration in the silenced leaves (12.1 μg/g FW) was less than that in the leaves of control lines (133.8 μg/g FW) (Fig. 7B). The PAs content in leaves from plantlets expressing with TRV-GFP-*McMYB12s* was approximately 70% lower than in the control lines (Fig. 7B). Moreover, levels of rutin, quercetin-3-*O*-rhamnoside, phlorizin and avicularin were lower than in control leaves.

qRT-PCR revealed that *McMYB12a* and *McMYB12b* transcript levels in the *McMYB12s*-silenced new buds decreased by approximately 90% of levels in the buds of control plantlets. Transcript levels of the McMYB12a and McMYB12b target genes, *McCHS, McANS, McANRs*, and *McLARs*, were significantly lower in the silenced lines than in the control lines. Furthermore, down-regulation of the expression of McMYB12a and McMYB12b target genes decreased the expression of other flavonoid biosynthetic genes, such as *McF3H, McF3’H, McDFR, McUFGT* and *McFLS* (Fig. 7C). The abundance of these gene transcripts was consistent with anthocyanin, PAs and flavonols levels and the degree of pigmentation observed in the *McMYB12s*-silenced and control lines (Fig. 7C).

To further confirm the function of *McMYB12a* and *McMYB12b* as flavonoid regulators, *Agrobacterium tumefaciens* containing the vectors *35 S::McMYB12* (pBI121, for overexpression) or pTRV2-*McMYB12* (TRV, for silencing) was injected into apple fruits, respectively. Interestingly, the apple fruits transformed with *35 S::McMYB12a* rapidly accumulated anthocyanins at the injection sites, resulting in significant red coloration (Fig. 8A), while overexpression of *McMYB12b* repressed fruit coloration, with the injection sites having a light green appearance, unlike the control peels (Fig. 8A). Suppression of *McMYB12a* and *McMYB12b* expression using a pTRV2-*McMYB12s* plasmid with a partial *McMYB12* ORF with identical sequence in McMYB12a and McMYB12b, inhibited anthocyanin accumulation and the peel was white at the injection sites (Fig. 8A).

HPLC analysis showed that overexpression of *McMYB12a* resulted in a significantly increase in anthocyanins accumulation and a small increase in PA contents, while a decrease in quercetin-3-*O*-rhamnoside in transgenic fruit than that in control lines. In contrast, the anthocyanin content of *McMYB12b* overexpressing apple fruit was lower than in the control fruit, while epicatechin and procyanidin B2 contents increased almost 2-fold. In pTRV2-*McMYB12* infiltrated peels, silencing of *McMYB12* caused a decrease in anthocyanin, PA and quercetin-3-*O*-rhamnoside abundances (Fig. 8B).

The transcript levels of the *McMYB12* genes and several flavonoid biosynthetic genes in the transformed apple fruit were evaluated using qRT-PCR. We observed that changes in the PAs and anthocyanin contents were accompanied by increased or reduced transcript levels of the closely related apple genes, *MdMYB121* (99% sequence identity to *McMYB12a*) and *MdMYB12* (99% sequence identity to *McMYB12b*), and the PAs biosynthesis genes (Fig. 8C). Around the injection sites, compared with the control, the expression of *MdCHS, MdDFR, MdANS, MdUFGT* and *MdANR* was significantly up-regulated by overexpression of *McMYB12a*, while the expression of *MdF3H, MdDFR, MdANS, MdUFGT, MdFLS, MdANR* and *MdLAR* was substantially up-regulated in the *McMYB12b*-overexpressing fruits. In *McMYB12*-silenced fruit peels, expression of *MdCHS, MdF3’H, MdDFR, MdANS, MdUFGT, MdFLS, MdANR* and *MdLAR* was strongly down-regulated compared to wild-type peels. We inferred from these results that the *McMYB12a* and *McMYB12b* may have different functions and target genes in the flavonoid biosynthetic pathway (Fig. 8C).

To further confirm the specific role of *McMYB12* genes in PAs biosynthesis pathway, we evaluated the expression of several other MYB transcription factors which involved flavonol and anthocyanins regulation in the gene silenced or overexpressed crabapple leaves or apple peels. qPCR results revealed that increased or decreased levels of expression of the *McMYB12* genes could alter the expression of *McMYB4* (a regulator of flavonol biosynthesis with 99% amino acids sequence similarity with MdMYB22) and *McMYB16* (a suppressor of anthocyanin biosynthesis with 99% amino acids sequence similarity with MdMYB16) in crabapple leaves, or *MdMYB22* and *MdMYB16* in apple peels. Meanwhile, the transcription level of *McMYB10* (a regulator of anthocyanin biosynthesis with amino acids 100% sequence identity to MdMYB10) and *MdMYB10* not correlate with the expression variation of *MYB12s* in crabapple leaves and apple peels (Supplemental Fig. 8B,C,D). To test whether McMYB12s can co-regulate flavonoids accumulation with *McMYB4* or *McMYB16* by binding to their promoter, we also examined the binding ability among the promoters of *McMYB4* and *McMYB16* with McMYB12s proteins using yeast one hybrid analysis. We noted that *LacZ* activity was not detected in yeast harboring pLacZi-*proMcMYB4*, pLacZi-*proMcMYB16* together with pJG4-5-*McMYB12a* and pJG4-5-*McMYB12b* (Supplemental Fig. 8E). Taken together, we further hypothesize that McMYB12 plays a role in MYB transcription factors expression via modulating the flavonoids biosynthesis gene indirectly, this could involve in the altered secondary metabolism.

## Discussion

Our data suggest that two highly homologous R2R3-MYB TFs (McMYB12a and McMYB12b) can co-regulate the PAs and anthocyanin biosynthesis, thereby revealing a new mechanism for the regulation of PAs biosynthesis.

### PAs and anthocyanins in crabapple leaves

PAs, the products of the flavonoid pathway, accumulate in a wide variety of plant tissues and play roles in many physiological and biochemical processes^47^. Together with anthocyanins and flavonols, which are also antioxidants and have beneficial effects for human health, the regulation of their biosynthesis has been studied in a diverse range of crops, such as apple^11^,^16^,^34^,^35^,^36^,^37^,^38^,^40^,^41^, grape^26^,^27^,^28^,^29^,^30^,^31^,^32^,^33^,^48^ and strawberry (*Fragaria* × *ananassa*)^49^,^50^, reflecting their importance in fruit and vegetable color and flavor as well as their health promoting attributes.

**Figure 1:**
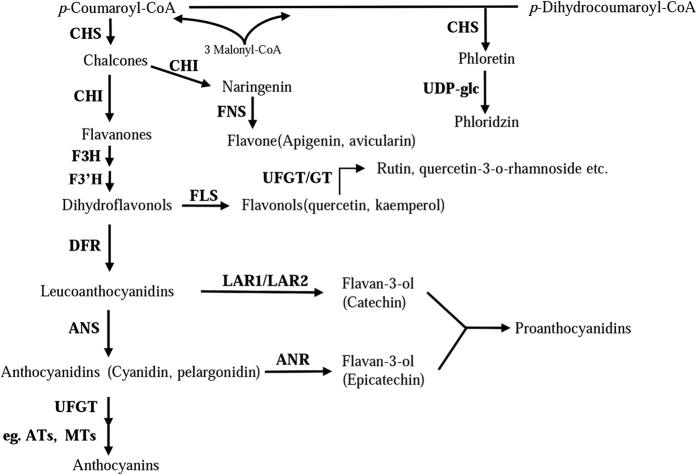
Schematic representation of the plant flavonoid biosynthetic pathway in apple. Enzymes required for flavonol, anthocyanin synthesis: CHS, chalcone synthase; CHI, chalcone isomerase; FNS, flavone synthase; F3H, flavanone 3-hydroxylase; F3′H, flavonoid 3′-monooxygenase; DFR, dihydroflavonol 4-reductase; ANS, anthocyanidin synthase; UFGT, UDP-glucose flavonoid 3-O-glucosyl transferase; FLS, flavonol synthase; LAR1/LAR2, leucoanthocyanidin reductase; ANR, anthocyanidin reductase. ATs, anthocyanin acyl transferases; MTs, anthocyanin methyl transferases.

**Figure 2:**
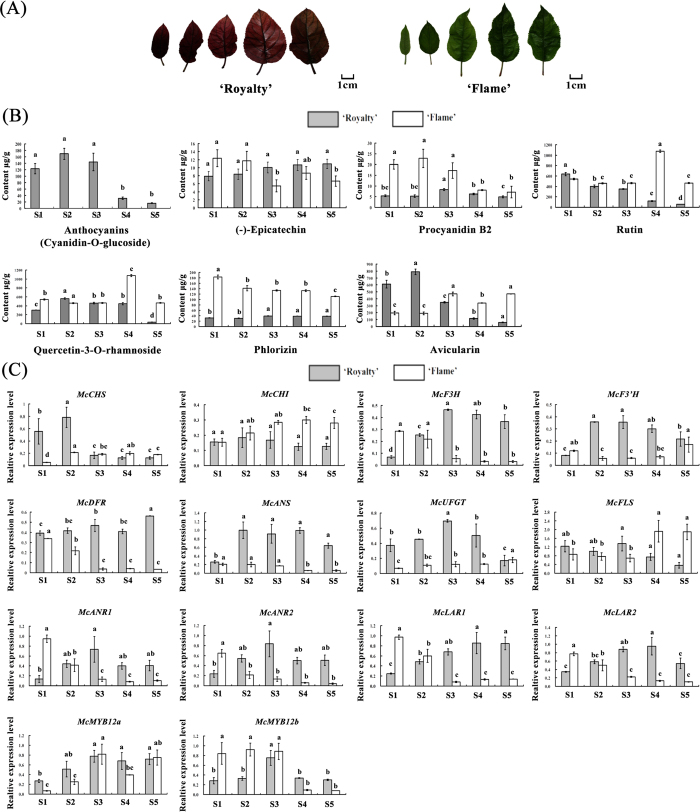
Analysis of flavonoid accumulation and expression profiles of flavonoid biosynthesis genes during 5 leaf development stages in the *Malus* crabapple ever-red cultivar ‘Royalty’ and ever-green cultivar ‘Flame’. (**A**) The five leaf developmental stages used for the analysis. (**B**) The content of the main flavonoids compounds in 5 leaf developmental stages of ‘Royalty’ and ‘Flame’. (**C**) Real-time PCR was used to analyze *McMYB12a, McMYB12b, McCHS, McCHI, McF3H, McF3’H, McDFR, McANS, McUFGT, McFLS, McANR-1, McANR-2, McLAR-1, McLAR-2* expression patterns in the leaves of ‘Royalty’ and ‘Flame’. *Malus 18 S* (DQ341382) was used as the reference gene. S1 to S5 represents stage 1, 2, 3, 4 and 5 of leaf development. Error bars indicate the standard error of the mean ± SE of three replicate measurements. The expression levels and correlation of flavonoid regulatory and biosynthetic genes were calculated using CFX-Manager-3-1 following the manufacturer’s instructions (Bio-Rad). Scale bars = 1 cm. Different letters above the bars indicate significantly different values (*P* < 0.05) calculated using one-way analysis of variance (ANOVA) followed by a Tukey’s multiple range test.

**Figure 3:**
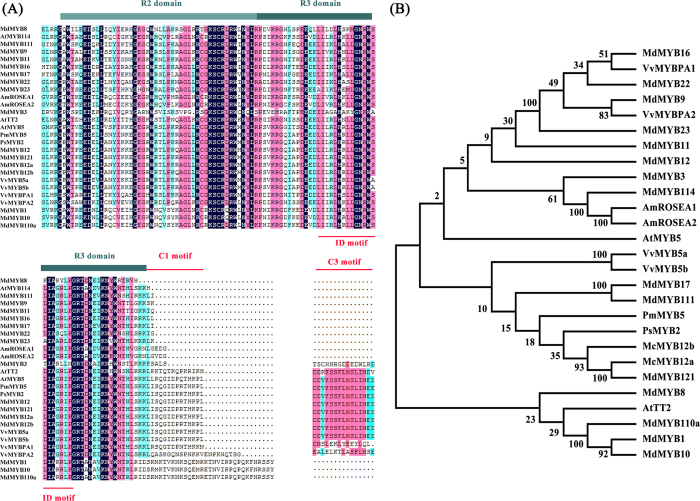
Deduced amino acids sequence characteristics of McMYB12 derived from ‘Royalty’ leaf RNA samples. (**A**) Sequence alignment of McMYB12 proteins and R2R3 MYB regulators from other species. The position of the R2R3-type MYB domain is indicated by cyan lines, while motif 1, involved in the interaction with bHLH proteins, and the C1 and C3 motifs are highlighted by red lines. Amino acids identical in all sequences are shaded black. (**B**) Phylogenetic analysis of *McMYB12a* and *McMYB12b* and MYB genes from other species involved in the regulation of PAs biosynthesis. The scale bar represents the number of substitutions per site and the numbers next to the nodes are bootstrap values from 1,000 replicates. Phylogenic and molecular evolutionary analysis was conducted using MEGA version 5.1. The GenBank database accession numbers of the proteins are as follows: AmROSEA1, ABB83826; AtMYB114, AT1G66380; AmROSEA2, ABB83827; AtMYB5, NP187963; AtMYB5, NM112200; AtTT2, Q9FJA2; PsMYB2, KJ466975; PmMYB5, XM008222439; VvMYB5a, AAS68190; VvMYB5b, NM001280925; VvMYBPA1, CAJ90831; VvMYBPA2, EU919682; MdMYB1, ABK58136; MdMYB3, HM122621; MdMYB8, DQ267899; MdMYB9, DQ267900; MdMYB10, ACQ45201; MdMYB11, DQ074463; MdMYB12, HM122616; MdMYB16, HM122617; MdMYB17, HM122618; MdMYB22, DQ074470; MdMYB23, DQ074471; MdMYB110a, JN711473; MdMYB111, HM122615; MdMYB121, KC834015; McMYB12a, KJ020112; McMYB12b, KJ020111.

**Figure 4:**
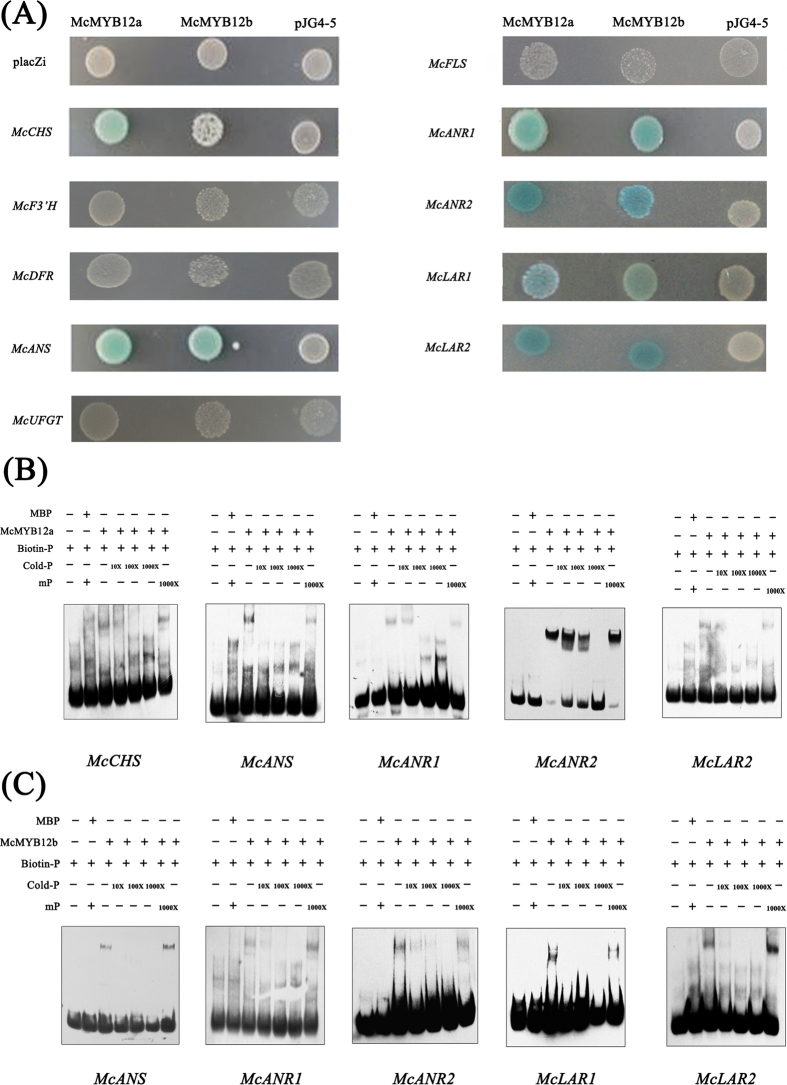
*Cis*-element binding ability and transcriptional activation assays of McMYB12a and McMYB12b with anthocyanin and PAs biosynthetic genes. (**A**) Interaction of McMYB12 proteins with the promoters of flavonoids biosynthetic genes as revealed by yeast one-hybrid assays. The panel shows yeast cells containing distinct effector and reporter constructs grown on an SD/-Trp/-Ura medium plate. Interaction of McMYB12a or McMYB12b, fused to the GAL4 activation domain (pJG4-5-*McMYB12a* or *b*), with *LacZ* driven by the flavonoid biosynthetic gene promoters (pLacZi-promoter of flavonoid biosynthetic gene) is shown in left top panel. Yeast transformed with pJG4-5-*McMYB12s*/pLacZi, pJG4-5/pLacZi-promoter of flavonoid biosynthetic genes and pJG4-5/pLacZi were used as controls. (**B**) Gel-shift analysis of McMYB12a binding to the promoters of six flavonoid biosynthetic genes. Purified proteins (10 μg) were incubated with 25 picomoles biotin-labeled probe or mutant probe (mP). For the competition test, non-labeled probes at varying concentrations (from 10- to 1,000-fold excess) or labeled mutant probes (1,000-fold) were added to the above experiment. (**C**) Gel-shift analysis of McMYB12b binding to the promoters of five flavonoid biosynthetic genes.

**Figure 5:**
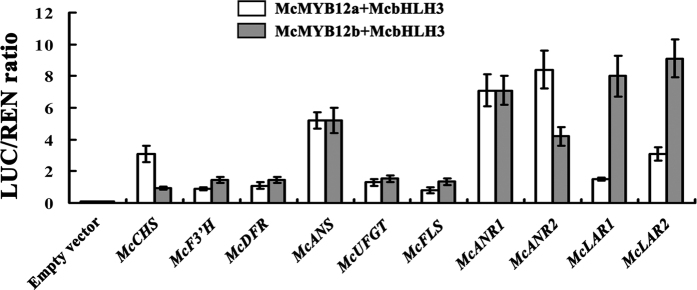
Trans-activation of the flavonoids biosynthesis gene promoters by McMYYB12a and McMYB12b in a dual luciferase transient assay. For each TF-promoter interaction, three independent experiments were performed (at least six replicates in each experiment). The ratio of LUC/REN of the empty vector (SK) plus promoter was used as calibrator (set as 1). Error bars indicate SEs from six biological replicates. Leaves of *Nicotiana benthamiana* were coinfiltrated with flavonoids biosynthesis gene promoters-*LUC, 35 S*::*McMYB12a* and *35 S*::*McbHLH3, 35 S*::*McMYB12b* and *35 S*::*McbHLH3*.

**Figure 6:**
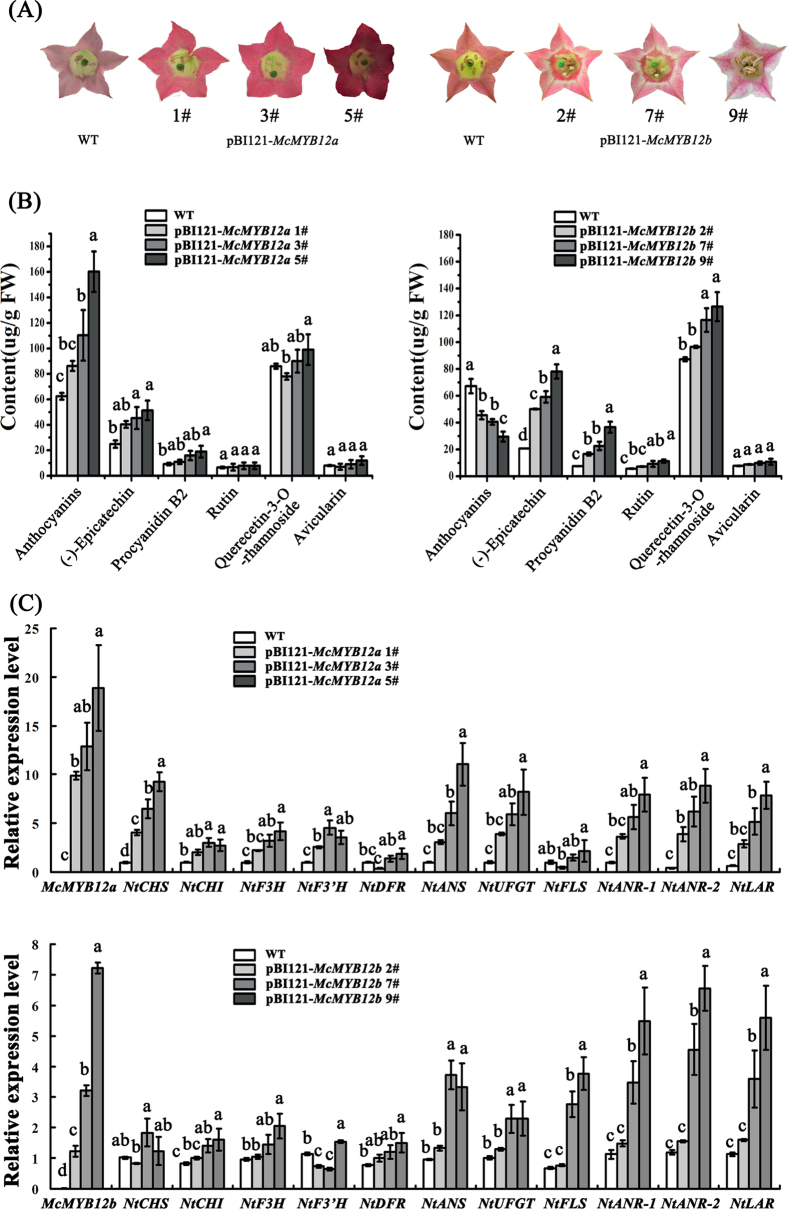
Overexpression of *McMYB12a* and *McMYB12b* in tobacco elevates different flavonoids production. (**A**) The phenotype of transgenic tobacco flowers over-expressing *McMYB12a* and *McMYB12b*, respectively. Flowers of pBI121-*McMYB12a* plants showed an increased pigmentation in petal compared to pBI121-*McMYB12b* and control flowers. And Flowers of pBI121-*McMYB12b* plants showed lighter pink than the control flowers. (**B**) The flavonoids compound concentrations in different samples. (**C**) qRT-PCR analysis of *McMYB12a* and *b* and anthocyanin pathway genes in the petals each of three different transformed *35 S::McMYB12s* tobacco lines. pBI121-*McMYB12a* 1#, pBI121-*McMYB12a* 3# and pBI121-*McMYB12a* 5#, three independent *McMYB12a*-overexpressing lines; pBI121-*McMYB12b* 2#, pBI121-*McMYB12b* 7# and pBI121-*McMYB12b* 9#, three independent *McMYB12b*-overexpressing lines. Error bars indicate the mean ± SE of three replicate reactions. Different letters above the bars indicate significantly different values (*P* < 0.05) calculated using one-way analysis of variance (ANOVA) followed by a Tukey’s multiple range test.

**Figure 7:**
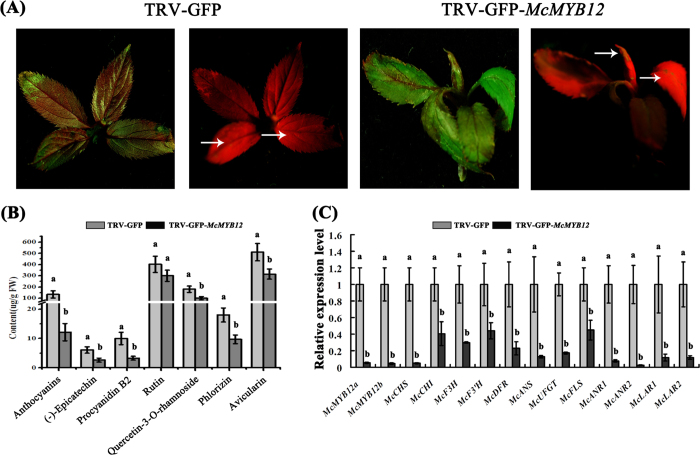
Silencing *McMYB12* genes in the ever-red crabapple cultivar ‘Royalty’. (**A**) The faded red leaf phenotype was visualized at 14 days post infiltration. (**B**) flavonoids compound levels in inoculated ‘Royalty’ plantlets in μg/g flesh weight. (**C**) Relative expression levels in inoculated ‘Royalty’ tissue culture plantlets were determined using qRT-PCR. Error bars indicate the standard error of the mean ± SE of three replicate measurements. Different letters above the bars indicate significantly different values (*P* < 0.05) calculated using one-way analysis of variance (ANOVA) followed by a Tukey’s multiple range test.

**Figure 8:**
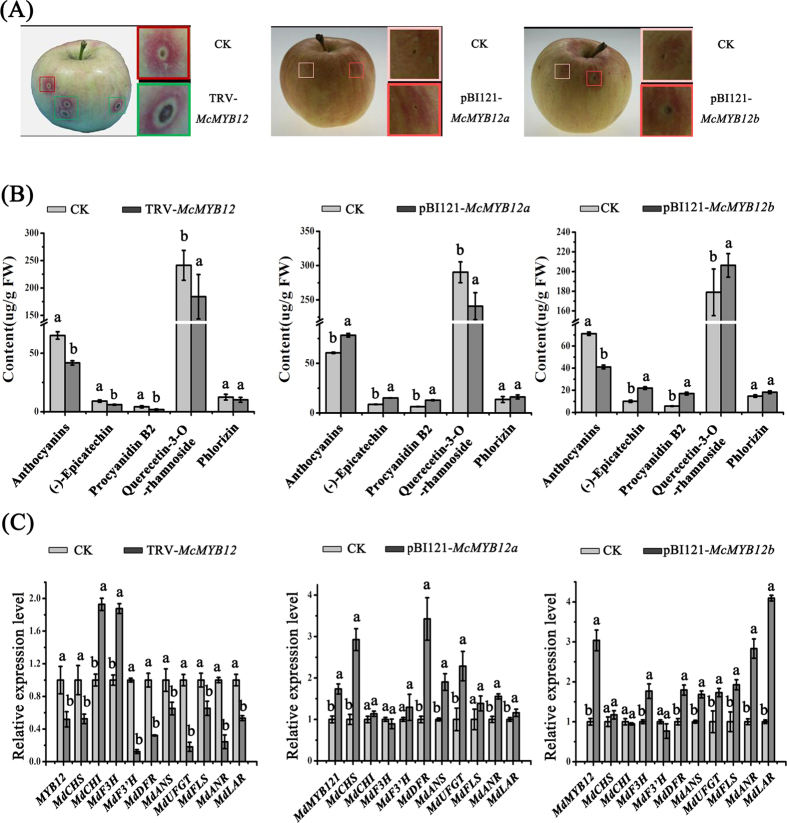
Transient expression of *McMYB12* genes in apple fruit. *McMYB12s* were overexpressed using the pBI121-*McMYB12a* and *b* vectors, or the gene was suppressed in apple fruit using the pTRV2-*McMYB12s* vector. Apple fruit injected with the empty pBI121 and TRV vectors and infiltration buffer only were used as controls. (**A**) Apple fruit peel coloration around the injection sites of overexpressing (pBI121-*McMYB12a* and *b*) or suppressed (pTRV2-*McMYB12s*) apples. (**B**) flavonoids compound levels around the injection sites of apple peels in μg/g flesh weight (FW). (**C**) Relative expression levels around the infiltration sites were determined using qRT-PCR, the expression level of MYB12 represent the transcription level of *MdMYB12* and *MdMYB121* in silenced fruits. *MdMYB121* have 99% sequence identity to *McMYB12a* and *MdMYB12* have 99% sequence identity to *McMYB12b*. Error bars indicate the standard error of the mean ± SE of three replicate measurements. Different letters above the bars indicate significantly different values (*P* < 0.05) calculated using one-way analysis of variance (ANOVA) followed by a Tukey’s multiple range test.

The biosynthetic pathways of PAs, anthocyanins and flavonols share common steps in the flavonoid pathway (from CHS to F3H), and each class of flavonoid (anthocyanin, flavonol, and flavan-3-ol) is synthesized by a multienzymatic step enzyme reaction branching from the common flavonoid pathway (Fig. 1)^11^. Several studies have shown that the competitive relationship between anthocyanins and flavonols is important for coloration^46^,^51^; however, further research is required to understand the association between PAs and anthocyanin accumulation. Our results indicate that crabapple leaf color is closely related to both anthocyanin and PAs abundance. In crabapple, a gradual decrease in anthocyanin content was observed in ‘Royalty’ leaves during their development, and the levels of PAs compounds showed an increasing trend relative to the decrease in anthocyanin accumulation. In contrast, because of the deficiency in anthocyanin biosynthesis, anthocyanin was almost undetectable in the leaves of the ‘Flame’ cultivar, and the PAs content was higher than that in ‘Royalty’ (Fig. 2). In addition, up-regulation in the expression of the PAs regulator *McMYB12b* significantly enhanced the accumulation of PAs, and inhibited the biosynthesis of anthocyanins (Figs 6A and 8). We deduced from these data that there is a metabolic balance between flavonol and anthocyanin biosynthesis^46^, and that the anthocyanin and PAs biosynthetic pathways have a competitive relationship in crabapple leaves. The massive expression of *McCHS*, an upstream enzyme in flavonoids biosynthesis, may promote the accumulation of anthocyanins in *McMYB12a*-overexpression lines. Meanwhile, as the target gene of McMYB12a and McMYB12b, *McLAR1* very likely play a key role in balance between anthocyanin and PAs production.

### Expression of *McMYB12* genes correlates with PAs synthesis in *Malus* crabapple

Several MYB TFs are known to be involved in the regulation of PAs biosynthesis. In grapevine, the expression of *VvMYBPA1* during flower and early berry development, and in seeds before ripening, correlates with PAs accumulation and the expression of PAs-specific biosynthetic genes^29^. The spatiotemporal expression of *VvMybPA2*, which is restricted to the skin of young berries and leaves, is congruent with a role in the regulation of PAs biosynthesis. Moreover, the expression of *VvMybPA2* in berry skins suggests that it is more likely than *VvMybPA1* to be involved in PAs synthesis^29^,^30^. The expression of *VvMYB5b*, combined with the action of specific regulators, such as *VvMYBA1* and *VvMYBPA1*, controls the biosynthesis of both anthocyanins and PAs throughout grape berry development^52^. Recently, a grape R2R3-MYB transcription factor, *VvMYBPAR*, was shown to be relatively highly expressed in the skins of young berries, with even higher levels in the seeds, and maximal expression was detected around the veraison developmental stage^31^. In apple, epicatechin and catechin biosynthesis is under the control of the biosynthetic enzymes ANR and LAR, respectively^16^. The expression of *MdMYB9* and *MdMYB11* followed a similar pattern as the biosynthetic enzyme MdANR, with high relative expression levels detected in flowers and old leaves for all genes^40^,^41^.

In the current study of the ‘Royalty’ and ‘Flame’ crabapple cultivars, we found that the expression patterns of the two *McMYB12a* and *McMYB12b* during leaf, petals and fruits development are consistent with them having a role in regulating PAs synthesis. The transcript profiles of *McMYB12a* and *McMYB12b* correlated with those of *McANRs* and *McLARs*, and with the accumulation of PAs, suggesting that the McMYB12 proteins may regulate PAs synthesis by modulating the expression of genes that are specific to the PAs branch of the phenylpropanoid pathway (Fig. 2C). Interestingly, we found that the expression of *McMYB12a* partially correlated with the accumulation of anthocyanins, so these TFs may affect not only just one or two branches of the phenylpropanoid pathway, but rather a large portion of the pathway (Fig. 2B,C), as was reported to be the case for *VvMYB5a*^32^ and *MdMYB9* and *MdMYB11*^41^. The patterns of anthocyanin and PAs accumulation showed a similar trend, so we conclude that the biosynthesis of both classes of compounds shares upstream genes/enzymes, and that their biosynthesis occurs concurrently.

Furthermore, we also investigated the promoter structures of *McMYB12s* in different crabapple cultivars, the results showed that there are no significantly structure difference and GUS activity variation of *McMYB12s* promoters between different crabapple cultivars (Supplemental Fig. 5). The different expression levels of *McMYB12*s in different crabapple cultivars maybe caused by transcriptional modification or post-transcriptional modification^36^,^53^.

### *McMYB12a* and *McMYB12b* are involved in the balance of PAs and anthocyanin biosynthesis in *Malus* crabapple

*MdMYB9* and *MdMYB11* which were induced by methyl jasmonate (MeJA) and stress conditions were functionally characterized. Protein interaction assays showed that both MYB proteins interact with MdbHLH3, and promoter binding assays showed both MdMYB9 and MdMYB11 bind to the promoters of *ANS, ANR* and *LAR*, whereas MdbHLH3 is recruited to the promoters of *MdMYB9* and *MdMYB11* and regulates their transcription^41^.

In the current study, yeast one hybrid, EMSA and transient activation assays showed that McMYB12a acts as the upstream regulator of *McCHS, McANS, McANR1, McANR2* and *McLAR2*, while *McANS, McANR1, McANR2, McLAR1* and *McLAR2* are the direct targets of McMYB12b. This difference in the downstream target genes of the McMYB12 proteins provided evidence for them having different functions. We therefore further hypothesize that McMYB12a acts together with McMYB12b to regulate PAs biosynthesis. Meanwhile, we speculated that McMYB12s may have patially function similarity with MdMYB9 and MdMYB11.

Variation in the concentrations of phenolic compounds (e.g. rutin, querecetin-3-*O*-rhamnoside, phlorizin, avicularin) was observed in *McMYB12s* -overexpressing or silenced organs. In addition, we observed a correlation between variation in expression of the *McMYB12* genes and the transcript levels of others MYB transcription factors which involved in flavonol and anthocyanins biosynthesis in crabapple leaves and apple peels. Moreover, the yeast one hybrid results showed that *LacZ* activities were not detected in yeast harboring pLacZi-*proMcMYB4*, pLacZi-*proMcMYB16* together with pJG4-5-*McMYB12a* and pJG4-5-*McMYB12b*. Based on these results, we speculated that variation in levels of these compounds occurs via the *McMYB12* genes directly or indirectly modulating the expression of the flavonoid biosynthesis genes. This might involve a variety of mechanisms, including redistribution of secondary metabolites/solutes, or altered secondary metabolism. We conclude that an important function of McMYB12 transcription factors is to regulate the biosynthesis of anthocyanins and PAs by binding to the promoters of downstream anthocyanin and PAs biosynthesis genes.

**Figure 9:**
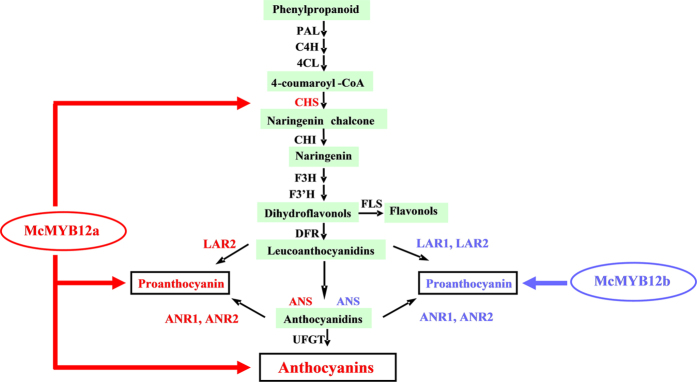
Schematic showing McMYB12a and McMYB12b have different functions by regulating different target genes in flavonoids pathway. The expression of *McMYB12a* significantly activating the expression of *McCHS, McANS, McANR1, McANR2* and *McLAR2*, leading to accumulation.of anthocyanin and PAs (Red). However, *McMYB12b* can regulate the transcription level of *McANS, McANR1, McANR2, McLAR1* and *McLAR2* and the accumulation of PAs in crabapple leaves.

The extant apple chromosomes homologies derived from a putative nine-chromosome ancestor^53^. Each doublet of the eight apple chromosomes (3–11, 5–10, 9–17 and 13–16) is derived principally from one ancestor, and interestingly, McMYB12a and McMYB12b are located on chromosomes 3 and 11, respectively. In the Rosaceae, including *Malus* spp., an evolutionary trend toward important trait specialization may have been partially based on gene duplication, resulting in the creation of large families of paralogous genes^53^. *McMYB12a* and *McMYB12b*, which share 92% sequence identity, regulate different biosynthetic genes involved in the modulation of anthocyanin and PAs levels in a counterbalanced secondary metabolism. This may account for phenotypic leaf color variation in crabapple. Heterologous and homologous expression analyses results showed that overexpression of *McMYB12a* affected the expression of various flavonoid structural genes, as well as the amounts of anthocyanins and PAs. In contrast, *McMYB12b* overexpression promoted the accumulation of PAs, but did not activate anthocyanin synthesis, suggesting it may be specific to the PAs pathway. Conversely, silencing of both *McMYB12a* and *McMYB12b* in crabapple plantlets suppressed the accumulation of anthocyanins and PAs and decreased the expression of anthocyanin and PAs biosynthetic genes. We propose that *McMYB12b* specifically regulates the PAs biosynthetic pathway, as is the case with *VvMYBPA1*, a transcriptional regulator of PAs synthesis in grape^29^, while McMYB12a is mainly responsible for regulating anthocyanin accumulation and partially responsible for PAs accumulation in crabapple leaves. The different functions of *McMYB12a* and *McMYB12b* reflect the regulation of different downstream flavonoid biosynthetic genes (Fig. 9).

In this current study, we observed that several flavonoid biosynthetic genes were activated by *McMYB12s*, and that this activation was accompanied by the accumulation of anthocyanins and PAs. Promoter binding assays showed that McMYB12a and McMYB12b can regulate different flavonoid biosynthetic genes in crabapple to control the accumulation of different phenolic compounds. And these results provided evidence of the different functions of McMYB12a and McMYB12b. We further hypothesize that McMYB12a acts together with McMYB12b to regulate PA biosynthesis, and that McMYB12a may be involved in the regulation of anthocyanin biosynthesis by interaction with other MYB TFs, such as McMYB10. This current study provides an important framework for the future investigation of functional interactions between MYB TFs and the co-regulation of flavonoid accumulation.

## Materials and Methods

### Plant materials and growth conditions

Five-year-old *Malus* cv. ‘Royalty’ and ‘Flame’ trees grafted on *Malus hupehensis* were planted in the Crabapple Germplasm Resources Nursery in the Beijing University of Agriculture. The leaves of ‘Royalty’ and ‘Flame’ were collected at five different developmental stages (1, 2, 3, 4, 5) (Fig. 2A). The petals and fruits of ‘Royalty’ and ‘Flame’ were collected at five different developmental stages (1, 2, 3, 4, 5) (Supplemental Figs 1A, 2A and 3A).

*Nicotiana tabacum* cv. W38 was used for overexpression experiments. The plants were grown in a greenhouse at 27 °C under 16-h-light/8-h-dark illumination. Transgenic plants were created using *Agrobacterium tumefaciens* strain GV3101 by suspension in the *Agrobacterium* solution. *Nicotiana benthamiana* was used to promoter activity and transient activation assays. The plants were grown in pots in a growth chamber at 23 °C with a 16 h/8 h photoperiod and ~60% relative humidity.

For the apple studies, bagged fruits were harvested 145 days after full bloom (DAFB) from adult trees of the ‘Red Fuji’ cultivar (*M. pumila* Milier). The skin of the fruits was peeled, together with less than 1 mm of cortical tissue, for anthocyanin measurements and other analyses^54^.

Explants of *Malus* cv. ‘Royalty’ were harvested from one-year-old branches before spring bud germination, cultured on Murashige and Skoog medium supplemented with 0.1 mg/L 6-Benzylaminopurine (6-BA) and 2 mg/L (2,4-dichlorophenoxy) acetic acid (2,4-D) at 22 °C with a 16 h light (200 μmol. s^−1^. m^−2^)/8 h dark period.

### HPLC analysis

Crabapple leaves, tobacco seed and tobacco leaves (approximately 0.8–1.0 g fresh weight) were extracted with 10 mL extraction solution (methanol: water: formic acid: trifluoroacetic acid = 70: 27: 2: 1) as previously described at 4 °C in the dark for 72 h, with shaking every 6 h^55^. The liquid was separated from the solid matrix by filtration through sheets of qualitative filter paper. The filtrate was then passed through 0.22-μm reinforced nylon membrane filters (Billerica, MA, USA). Trifluoroacetic acid: formic acid: water (0.1: 2: 97.9) was used as mobile phase A, and trifluoroacetic acid: formic acid: acetonitrile: water (0.1: 2: 48: 49.9) was used as mobile phase B. PAs were extracted using 1 ml of 70% (v/v) acetone containing 0.1% (w/v) ascorbate, and incubated at room temperature for 24 h in the dark. The extract was centrifuged at 12, 000 g for 15 min at room temperature, and the supernatant was transferred to a new 1.5 ml microfuge tube. An aliquot of 200 μl of extract was dried at 35 °C and resuspended in 100 μl of 1% (v/v) HCl/methanol and 100 μl of 200 mM sodium acetate (pH 7.5). The injection volumes were 20 μl for anthocyanin and flavonol analysis or 10 μl for PA analysis. The gradients used were as follows: 0 min, 30% B; 10 min, 40% B; 50 min, 55% B; 70 min, 60% B; 80 min, 30% B. Detection was performed at 520 nm for anthocyanins, 350 nm for flavonols and 280 nm for PAs using an HPLC1100-DAD system (Agilent Technologies, Waldbronn, Germany)^56^. All samples were analyzed in biological triplicate.

### Cloning and sequence analysis of the full-length *McMYB12a* and *McMYB12b* genes

Total RNA was extracted from crabapple leaves using an RNA Extraction Kit (Aidlab, Beijing, China) according to the manufacturer’s instructions. The full-length cDNAs of *McMYB12a* and *McMYB12b* were amplified using the Rapid Amplification of cDNA Ends (RACE) method and nested PCR performed according to the manufacturer’s recommendations (Clontech, Palo Alto, CA). All PCR products were sub-cloned into the pGEM T-Easy Vector (Promega, Madison, WI) and transformed into *Escherichia coli* DH5α cells and sequenced. PCR primer sequences are listed in Table S1. Comparison and analysis of the sequences were performed using the advanced basic local alignment search tool (BLAST) at the National Center for Biotechnological Information (http://www.ncbi.nlm.nih.gov). The full-length DNA and protein sequences were aligned using DANMAN 5.2.2 (Lynnon Biosoft, USA). Phylogenetic and molecular evolutionary analyses were conducted with MEGA version 5.1, using a minimum evolution phylogeny test with a 1,000 bootstrap replicates^57^.

### Cloning and GUS activity analysis of the *McMYB12s* promoters

To analyze the differences in *McMYB12s* promoter sequences between the different cultivars, genomic DNA was isolated from leaves using the Plant Genomic DNA Kit (TIANGEN BIOTECH CO., LTD, Beijing, China). Cloning primers PMYB12a-F, PMYB12a-R PMYB12b-F and PMYB12b-R were designed using NCBI Primer BLAST and are listed in Table S1. PCR products were cloned into the pMD-19T vector and sequenced.

The promoter of *McMYB12a* and *McMYB12b* were cloned into the modified pBI121 vector^44^ using the *Xho*I and *Xba*I sites, and *Agrobacterium* GV3101 carrying *McMYB12a* promoter-*GUS* or *McMYB12b* promoter-*GUS* were collected by centrifugation at 4000 rpm for 10 min, and suspended in the infiltration buffer (10 mM MgCl_2_, 150 mM acetosyringone, and 10 mM MES, pH 5.6) to a final optical density at 600 nm of 0.8. CaMV 35 S promoter -*GUS* was as control. 100 μl bacterial suspension was infiltrated into intercellular spaces of a young tobacco leaf using a 1 ml plastic syringe without needle^58^. 2–4 pieces fully expanded young leaves were infiltrated per plant in three plants. After agroinfiltration, tobacco plants continued growing at 23 °C for 3 days as usual. Six technical replicates of 3 mm diameter leaf discs (about 1/3 of infiltrated area) were excised from each plant using a leaf hole-punch and buffered in Phosphate Buffer Saline (PBS). Subsequently, discs were respectively used for the histochemical GUS analysis and GUS activity assay^59^.

### qRT-PCR analysis

Total RNA from crabapple leaves, tobacco petals and apple peel were extracted as described above. DNase I (TaKaRa, Ohtsu, Japan) was added to remove genomic DNA, and the samples were then subjected to cDNA synthesis using the Access RT-PCR System (Promega, USA), according to the manufacturer’s instructions. The expression levels of flavonoid biosynthetic genes in crabapple and tobacco were analyzed using qRT-PCR and the SYBR Green qPCR Mix (TaKaRa, Ohtsu, Japan) and the Bio-Rad CFX96 Real-Time PCR System (BIO-RAD, USA), according to the manufacturers’ instructions. The PCR primers were designed using NCBI Primer BLAST and are listed in Table S1.

qRT-PCR analysis was carried out in a total volume of 20 μl containing 9 μl of 2 × SYBR Green qPCR Mix (TaKaRa, Ohtsu, Japan), 0.1 μM specific primers (each), and 100 ng of template cDNA. The reaction mixtures were heated to 95 °C for 30 s, followed by 39 cycles at 95 °C for 10 s, 50–59 °C for 15 s, and 72 °C for 30 s. A melting curve was generated for each sample at the end of each run to ensure the purity of the amplified products. The transcript levels were normalized using the *Malus 18 S ribosomal RNA* gene (DQ341382, for apple and crabapple) or the *NtActin* gene (GQ339768, for tobacco) as the internal controls and calculated using the 2^(−∆∆Ct)^ analysis method^60^.

### Overexpression in tobacco

The full length *McMYB12a* and *McMYB12b* open reading frames (ORFs) were cloned into the pBI121 vector^59^ using the *Bam*HI and *Sac*I sites, and *A. tumefaciens* strains carrying these constructs were used to transform *Nicotiana tabacum* ‘W38’ using the leaf disk method^61^. The PCR primers used are listed in Table S1. Transgenic plants were selected by kanamycin resistance. Two independent lines from the T2 progeny were used to compare color changes with the untransformed wild-type line.

### DMACA Staining of PAs

The presence of PAs in plant tissue was detected by staining the tissues with DMACA (dimethylaminocinnamaldehyde) solution (1% DMACA, 1% 6 N HCl in methanol). Dried seeds were stained for 6 h and the seedlings for 20 min. After staining, the samples were transferred to distilled water and blue staining was visualized and photographed with a Zeiss Discovery V20 stereomicroscope.

### Transient expression assays in crabapple plantlets and apple fruit

Fragments for the pTRV2-GFP-*McMYB12s* (458 bp) constructs were amplified by PCR with gene-specific primers, from a cDNA library derived from *Malus* crabapple leaves (cv. ‘Royalty’), using Taq DNA polymerase (TaKaRa, Ohtsu, Japan), according to the manufacturer’s instructions. The PCR primers used are shown in Table S1.

*A. tumefaciens* cells were grown, collected, and resuspended in a 10 mM MES, 10 mM MgCl_2_, and 200 mM acetosyringone solution to a final optical density of 1.5 at 600 nm and then incubated at room temperature for 3 to 4 h without shaking. Before infiltration, *A. tumefaciens* cultures containing combinations of pTRV1 (acts as an assistant vector and is responsible for virus replication and for allowing systemic movement throughout the host)^62^, pTRV2-GFP or its derivatives were mixed in a 1:1 ratio.

The infiltration protocol and culture methods for transient expression assays in crabapple plantlets were adapted as previously described^63^,^64^. The infiltration protocol and culture methods for apple fruits were adapted as previously described^46^.

For visualization of the GFP fluorescence in whole crabapple plants, the samples were illuminated with a 100 W hand-held long-wave ultraviolet lamp (UV products, Upland, CA, USA; Black Ray model B 100AP/R) and were photographed using a Kodak wratten filter 15.

### Yeast one-hybrid assay

A yeast one-hybrid system was used to assay the transcriptional activation by the McMYB12 proteins. The open reading frame of *McMYB12a* and *McMYB12b* were cloned into the *EcoRI* and *XhoI* sites of the pJG4-5 vector (Clontech, Palo Alto, CA, USA) under the control of the galactokinase 1 (GAL1) promoter as the effector constructs, respectively. The *McCHS, McANS, McANR-1, McANR-2, McLAR-1* and *McLAR-2* promoter sequences were inserted upstream of the reporter *LacZ* gene in the pLacZi vector. The effector and reporter or control constructs were transformed into competent cells of the yeast strain EGY48, resulting in the following yeast strains: pJG4-5-McMYB12s/pLacZi-promoter of flavonoid biosynthetic genes, pJG4-5/pLacZi-pro of flavonoid biosynthetic genes, pJG4-5-McMYB12s/pLacZi, pJG4-5/pLacZi. The cells were selected on synthetic drop-out media lacking tryptophan and uracil, and positive colonies were spotted onto glucose plates (2%) containing X-gal at 28 °C for 2 days to confirm blue color development^17^.

### EMSA

The *McMYB12a* and *McMYB12b* open reading frame sequences were cloned into the pMAL-C2X expression vector and transformed into Rosetta (DE3) *E. coli* competent cells. The maltose binding protein (MBP)-tag encoded in the pMAL-C2X vector was used to facilitate purification of the recombinant proteins. Isopropyl β-D-1-thiogalactopyranoside (IPTG) (0.3 mM) was added to the cultures induce *McMYB12a* or *McMYB12b* expression at 170 rpm for 6 h at 28 °C. The recombinant protein was purified with the One-Stop MBP-Tagged Protein Miniprep Pack (BioLab. Co. Ltd, Beijing, China). EMSA reactions were prepared according to the manufacturer’s protocol (LightShift^®^ Chemiluminescent EMSA Kit; Thermo Fisher Scientific). The probes were labeled by annealing biotin-labeled oligonucleotides. The oligonucleotides used for EMSA are listed in Table S2. Approximately 10 μg of purified McMYB12a or McMYB12b recombinant protein was used for each EMSA reaction.

### Dual luciferase assay of transiently transformed *Nicotiana benthamiana* leaves

The promoter fragments of flavonoids biosynthesis genes inserted into the cloning site of pGreenII 0800-LUC^65^, and modified to introduce an *Nco*I site at the 3′ end of the sequence, allowing the promoter to be cloned as a transcriptional fusion with the firefly *Luciferase* gene (*LUC*). In the same construct, a luciferase gene from *Renilla (REN*) under the control of a 35 S promoter provided an estimate of the extent of transient expression. *Agrobacterium* of the promoter–LUC fusion in pGreenII 0800-LUC (100 μL) was mixed with 300 μL each of two TF gene as effector cassette, 35S-*McMYB12a* or 35S-*McMYB12b* fusion in pART27 vectors, with *35 S::McbHLH3* gene in pGreenII 62-SK, respectively^11^. *N. benthamiana* plant growing conditions, *A. tumefaciens* infiltration processes, and luminescence measurements were as described previously^65^. For each TF-promoter interaction, three independent experiments were performed (at least six replicates in each experiments).

### Bimolecular fluorescence complementation assay

Bimolecular fluorescence complementation (BiFC) assay *in vivo* was performed as described^66^. McMYB12a, McMYB12b and McbHLH3 full-length sequence were amplified and cloned into the plasmid pSPYCE-35S/pUC-SPYCE and pSPYNE-35S/pUC-SPYNE, respectively. The pairs of the resulting fusion protein with corresponding empty vector were used as negative controls. These vectors were introduced into the *Agrobacterium* strain GV3101. The *Agrobacterium* were grown overnight in YEB media with appropriate antibiotic selection. *Agrobacterium* cultures containing the BiFC constructs plasmid were mixed at OD600 of 0.5: 0.5. Cells were pelleted by centrifugation and resuspended in infiltration medium (10 mM MgCl2, 10 mM MES, and 100 mM acetosyringone). After incubation for at least 2 h at room temperature, the suspension was infiltrated into leaves of 1-month old tobacco (*Nicotiana benthamiana*) plants. The photographs of YFP fluorescence were taken after 24–72 h of transformation using a confocal laser-scanning microscope (FV1000, Olympus, Tokyo, Japan).

## Additional Information

**How to cite this article:** Tian, J. *et al*. McMYB12 Transcription Factors Co-regulate Proanthocyanidin and Anthocyanin Biosynthesis in *Malus* Crabapple. *Sci. Rep.* **7**, 43715; doi: 10.1038/srep43715 (2017).

**Publisher's note:** Springer Nature remains neutral with regard to jurisdictional claims in published maps and institutional affiliations.

## Supplementary Material

Supplemental Figures and Tables
