# Supplementary material for: McMYB12 Transcription Factors Co-regulate Proanthocyanidin and Anthocyanin Biosynthesis in Malus Crabapple
Source: Sci Rep. 2017 Mar 3;7:43715. doi: 10.1038/srep43715 (PMC5334656; doi:10.1038/srep43715)
Supplement: Supplemental Figures and Tables [file srep43715-s1.doc]

**McMYB12 Transcription Factors Co-regulate** **Proanthocyanidin and Anthocyanin Biosynthesis in *Malus* Crabapple**

Ji Tian^a, b, c, d^, Jie Zhang ^a, b, c, d^, Zhen-yun Han^b, c, d^, Ting-ting Song ^b, c, d^, Jin-yan Li ^b, c, d^, Ya-ru Wang ^b, c, d^, Yun-cong Yao ^b, c, d^ *

^a^ Contributed equally to this work.

^b^ Department of Plant Science and Technology, Beijing University of Agriculture, Beijing, China.

^c^ Key Laboratory of New Technology in Agricultural Application of Beijing, Beijing University of Agriculture, Beijing, China.

^d^ Beijing Collaborative innovation center for eco-environmental improvement with forestry and fruit trees

*Correspondence should be addressed to Yun-cong Yao (Email: yaoyc_20@126.com; Tel.: +86-10-80799000)

**Supplemental Figures**

**
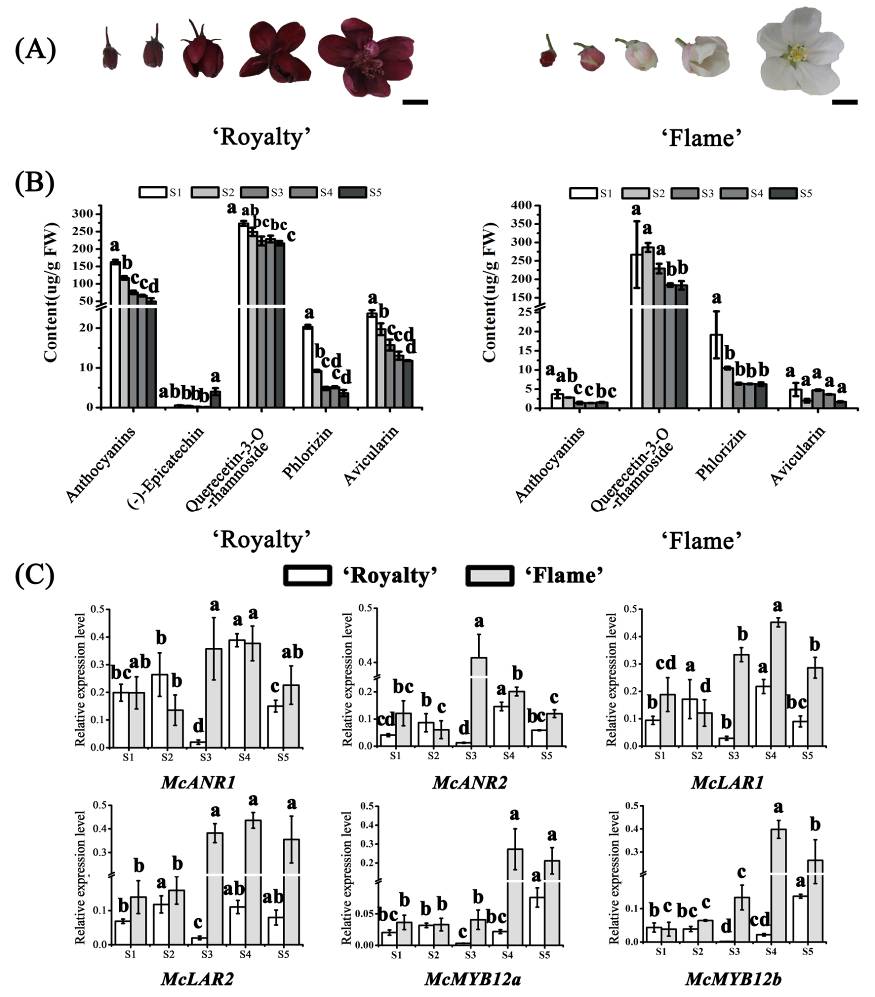
**

**Supplemental Figure** **1. Analysis of flavonoid accumulation and expression profiles of PAs biosynthesis genes during 5 petal development stages in the *Malus* crabapple ever-red cultivar ‘Royalty’ and ever-green cultivar ‘Flame’.** (A) The five petal developmental stages used for the analysis. (B) The content of the main flavonoids compounds (PAs) in 5 petal developmental stages of ‘Royalty’ and ‘Flame’. (C) Real-time PCR was used to analyze *McMYB12a*, *McMYB12b*, *McANR-1*, *McANR-2*, *McLAR-1*, *McLAR-2* expression patterns in the petals of ‘Royalty’ and ‘Flame’. *Malus* *18S* (DQ341382) was used as the reference gene. S1 to S5 represents stage 1, 2, 3, 4 and 5 of petal development. Error bars indicate the standard error of the mean ± SE of three replicate measurements. The expression levels and correlation of flavonoid regulatory and biosynthetic genes were calculated using CFX-Manager-3-1 following the manufacturer’s instructions (Bio-Rad). Scale bars=1cm. Different letters above the bars indicate significantly different values (*P*<0.05) calculated using one-way analysis of variance (ANOVA) followed by a Tukey’s multiple range test.

**
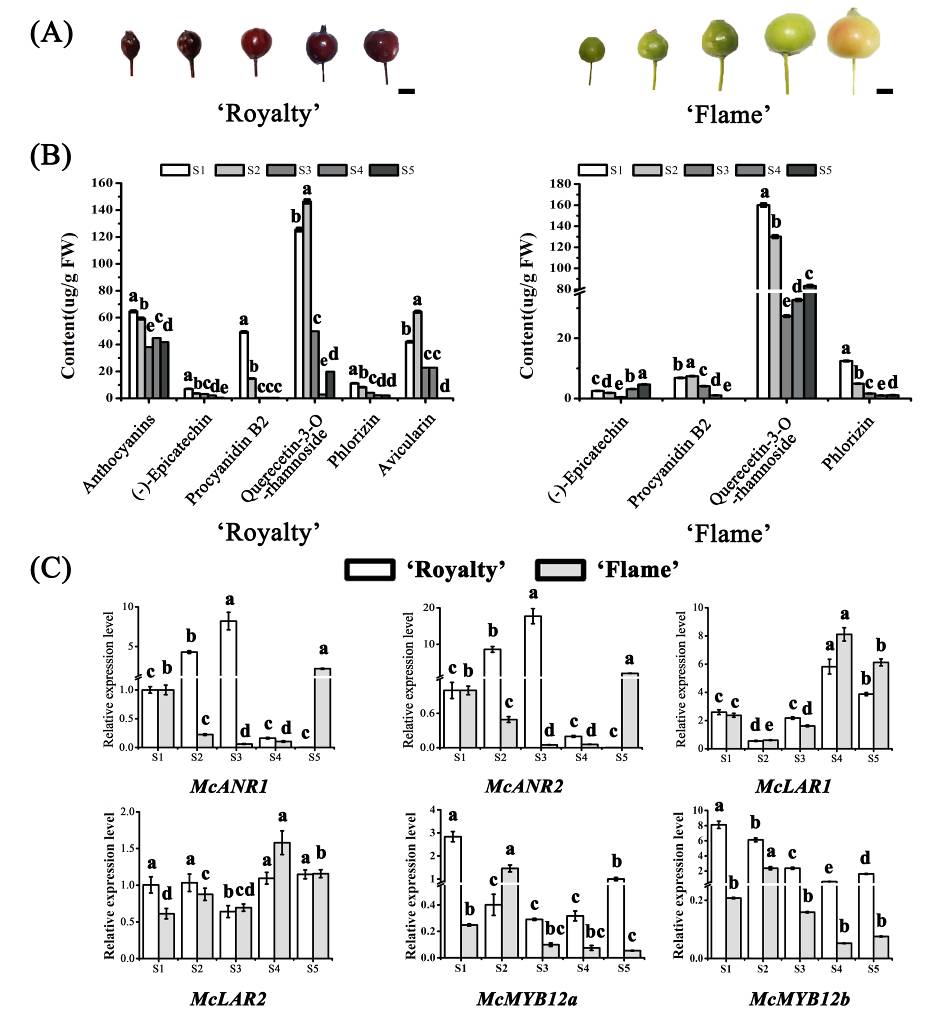
**

**Supplemental Figure** **2. Analysis of flavonoid accumulation and expression profiles of PAs biosynthesis genes during 5 fruit peel development stages in the *Malus* crabapple ever-red cultivar ‘Royalty’ and ever-green cultivar ‘Flame’.** (A) The five fruit peel developmental stages used for the analysis. (B) The content of the main flavonoids compounds in 5 fruit peel developmental stages of ‘Royalty’ and ‘Flame’. (C) Real-time PCR was used to analyze *McMYB12a*, *McMYB12b*, *McANR-1*, *McANR-2*, *McLAR-1*, *McLAR-2* expression patterns in the fruit peel of ‘Royalty’ and ‘Flame’. *Malus* *18S* (DQ341382) was used as the reference gene. S1 to S5 represents stage 1, 2, 3, 4 and 5 of fruit peel development. Error bars indicate the standard error of the mean ± SE of three replicate measurements. The expression levels and correlation of flavonoid regulatory and biosynthetic genes were calculated using CFX-Manager-3-1 following the manufacturer’s instructions (Bio-Rad). Scale bars=1cm. Different letters above the bars indicate significantly different values (*P*<0.05) calculated using one-way analysis of variance (ANOVA) followed by a Tukey’s multiple range test.

**
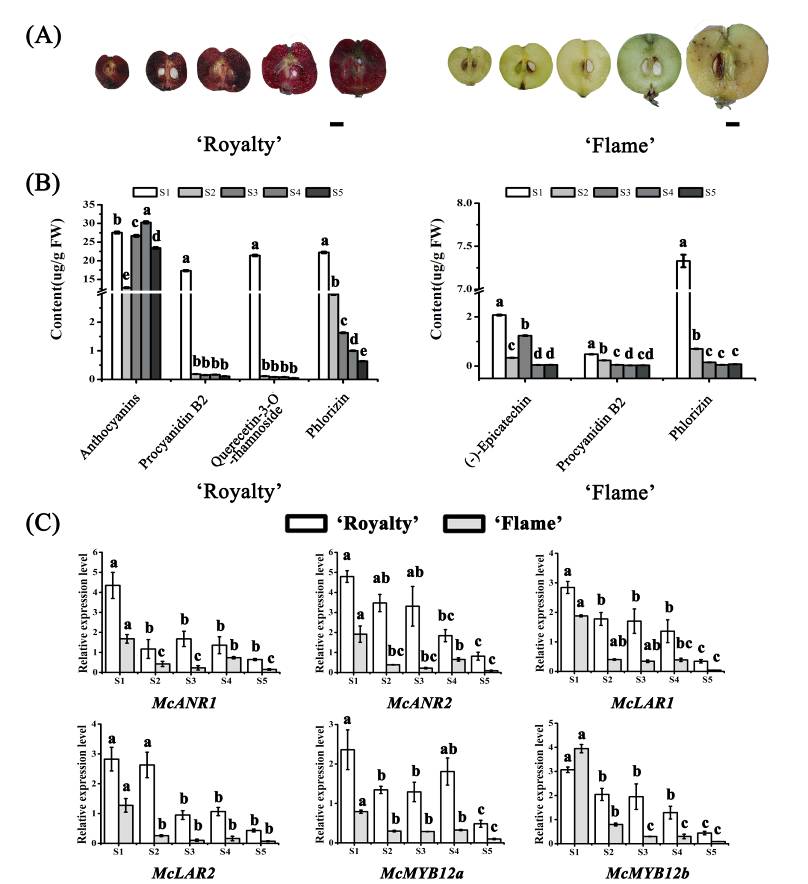
**

**Supplemental Figure** **3. Analysis of flavonoid accumulation and expression profiles of PAs biosynthesis genes during 5 fruit flesh development stages in the *Malus* crabapple ever-red cultivar ‘Royalty’ and ever-green cultivar ‘Flame’.** (A) The five fruit flesh developmental stages used for the analysis. (B) The content of the main flavonoids compounds (PAs) in 5 fruit flesh developmental stages of ‘Royalty’ and ‘Flame’. (C) Real-time PCR was used to analyze *McMYB12a*, *McMYB12b*, *McANR-1*, *McANR-2*, *McLAR-1*, *McLAR-2* expression patterns in the fruit flesh of ‘Royalty’ and ‘Flame’. *Malus* *18S* (DQ341382) was used as the reference gene. S1 to S5 represents stage 1, 2, 3, 4 and 5 of fruit flesh development. Error bars indicate the standard error of the mean ± SE of three replicate measurements. The expression levels and correlation of flavonoid regulatory and biosynthetic genes were calculated using CFX-Manager-3-1 following the manufacturer’s instructions (Bio-Rad). Scale bars=1cm. Different letters above the bars indicate significantly different values (*P*<0.05) calculated using one-way analysis of variance (ANOVA) followed by a Tukey’s multiple range test.

**
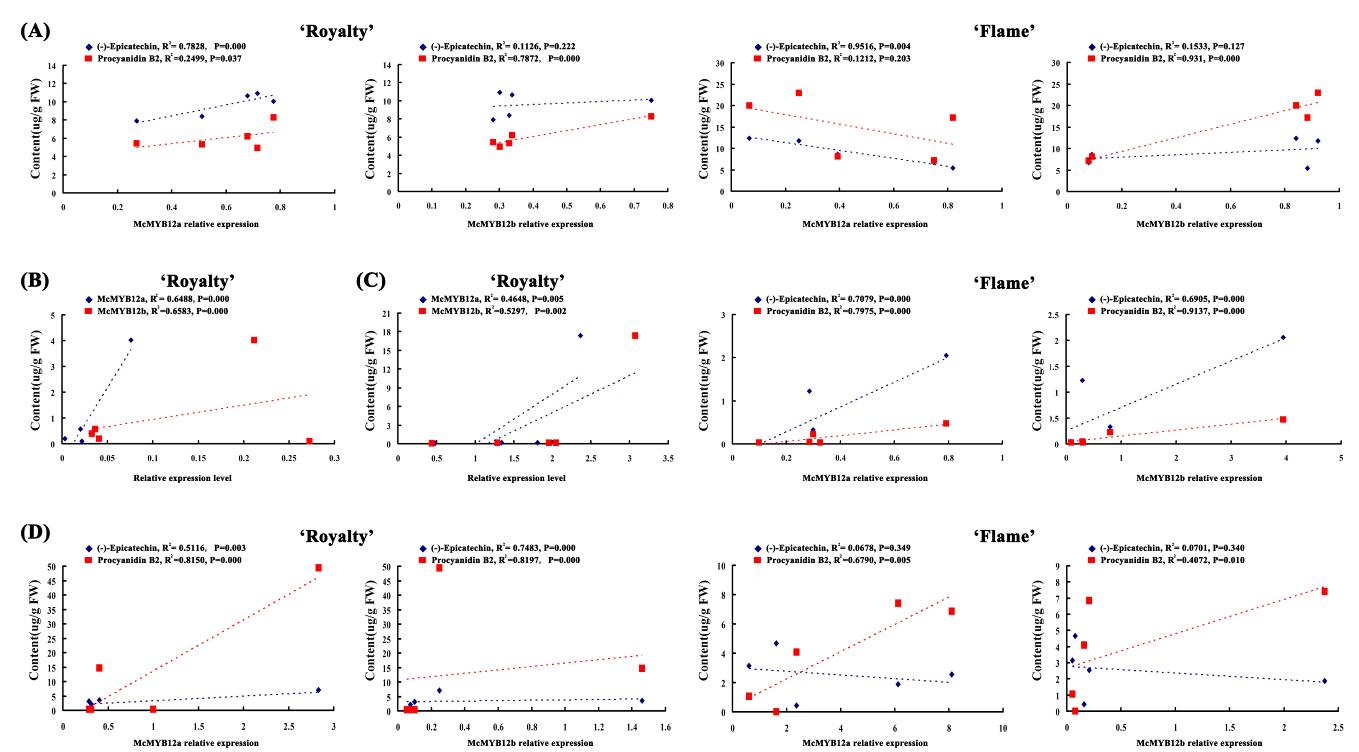
**

**Supplemental Figure** **4. Correlation analysis between relative expression levels of *McMYB12s* and the accumulation of PAs in different crabapple organs.** (A) Correlation analysis between relative expression levels of *McMYB12a*, *McMYB12b* and the accumulation of (-)-epicatechin and procyanidin B2 in crabapple leaves. (B) Correlation analysis between relative expression levels of *McMYB12a*, *McMYB12b* and the accumulation of (-)-epicatechin B2 in crabapple petals. (C) Correlation analysis between relative expression levels of *McMYB12a*, *McMYB12b* and the accumulation of (-)-epicatechin and procyanidin B2 in crabapple fruit flesh. (D) Correlation analysis between relative expression levels of *McMYB12a*, *McMYB12b* and the accumulation of (-)-epicatechin and procyanidin B2 in crabapple fruit peels.

**
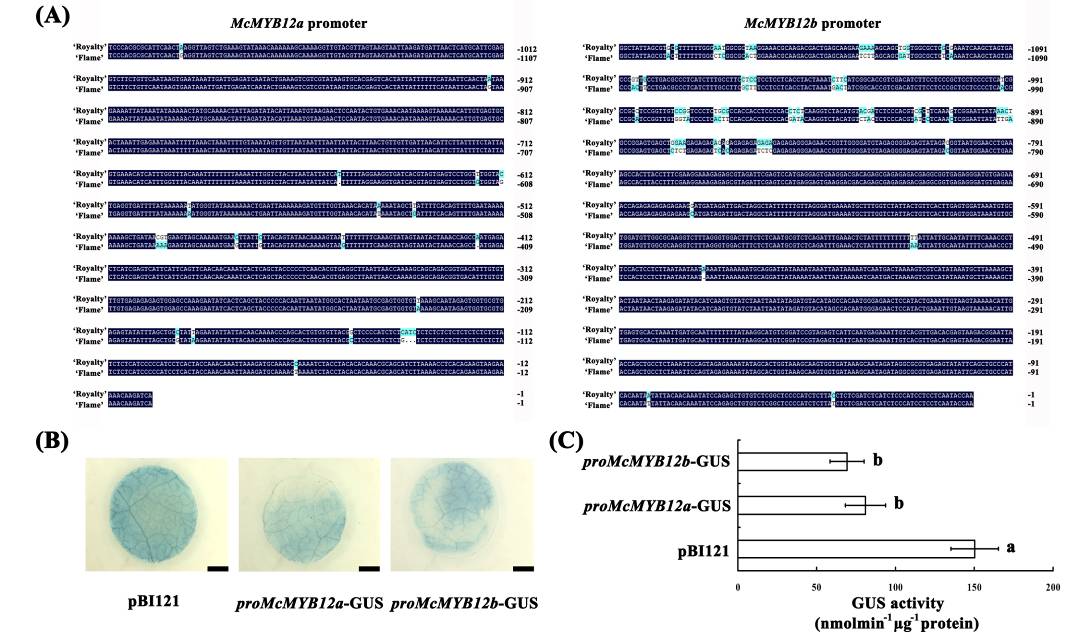
**

**Supplemental Figure 5. Promoter analysis of *McMYB12a* and *McMYB12b*.** (A) Promoter sequence alignments of *McMYB12a* and *McMYB12b* in ‘Royalty’ and ‘Flame’. (B) Histochemical GUS staining of discs from tobacco leaves infiltrated with *ProMcMYB12a*: *GUS* or *ProMcMYB12b*: *GUS*. CaMV 35S promoter -*GUS* (pBI121) was as control. No significant differences were observed between the promoter of *McMYB12a* and *McMYB12b* by GUS staining in tobacco leaves. (C) Quantitative GUS activity assay of discs from tobacco leaves infiltrated with *ProMcMYB12a*: *GUS* or *ProMcMYB12b*: *GUS*. Error bars represent SD of 6 biological replicates. Different letters above the bars indicate significantly different values (*P*<0.05) calculated using one-way analysis of variance (ANOVA) followed by a Tukey’s multiple range test.

**
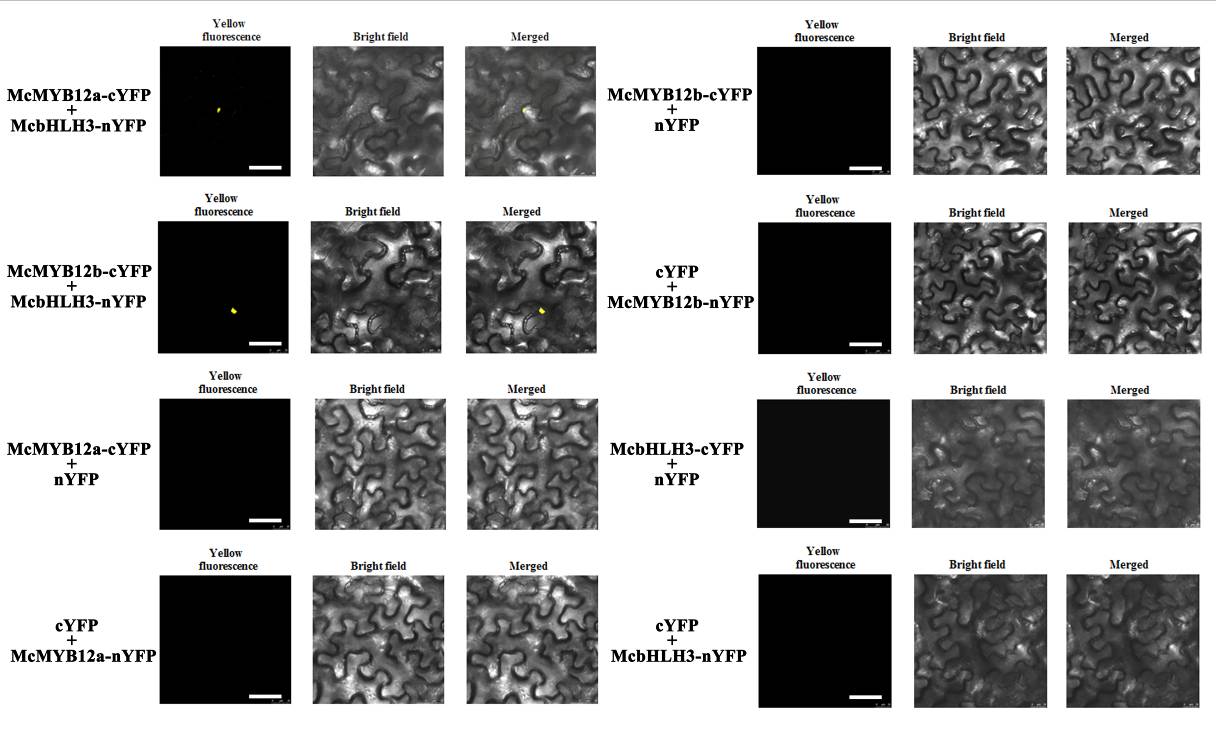
**

**Supplemental Figure** **6. BiFC visualization in *Agrobacterium* infiltrated tobacco leaves.** YFP fluorescence, bright field, and merged images are shown for each transformation combination. The construct pairs of McMYB12a-cYFP, McMYB12a-nYFP, McMYB12b-cYFP, McMYB12b-nYFP, McbHLH3-cYFP, McbHLH3-nYFP with empty vector, respectively, were used as negative controls. Bars = 20 μm.

**
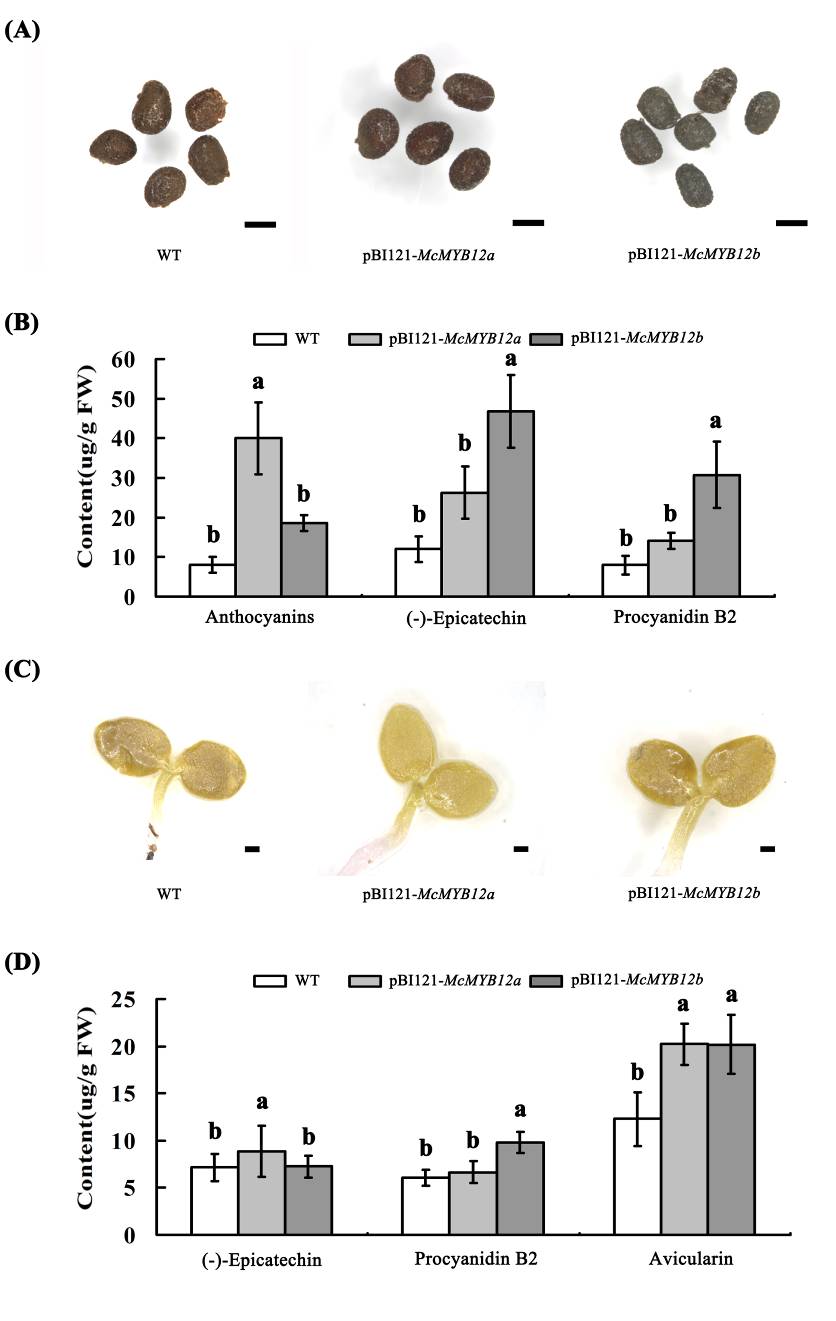
**

**Supplemental Figure** **7. Accumulation of PAs compounds in McMYB12s overexpressing tobacoo seeds.** (A) DMACA staining of tobacco seeds from transgenic lines and control lines (B) The flavonoids compound concentrations in transgenic and control tobacco seeds. (C) DMACA staining of tobacco leaves from transgenic lines and control lines (D) The flavonoids compound concentrations in transgenic and control tobacco leaves. Scale bars=0.5 mm.


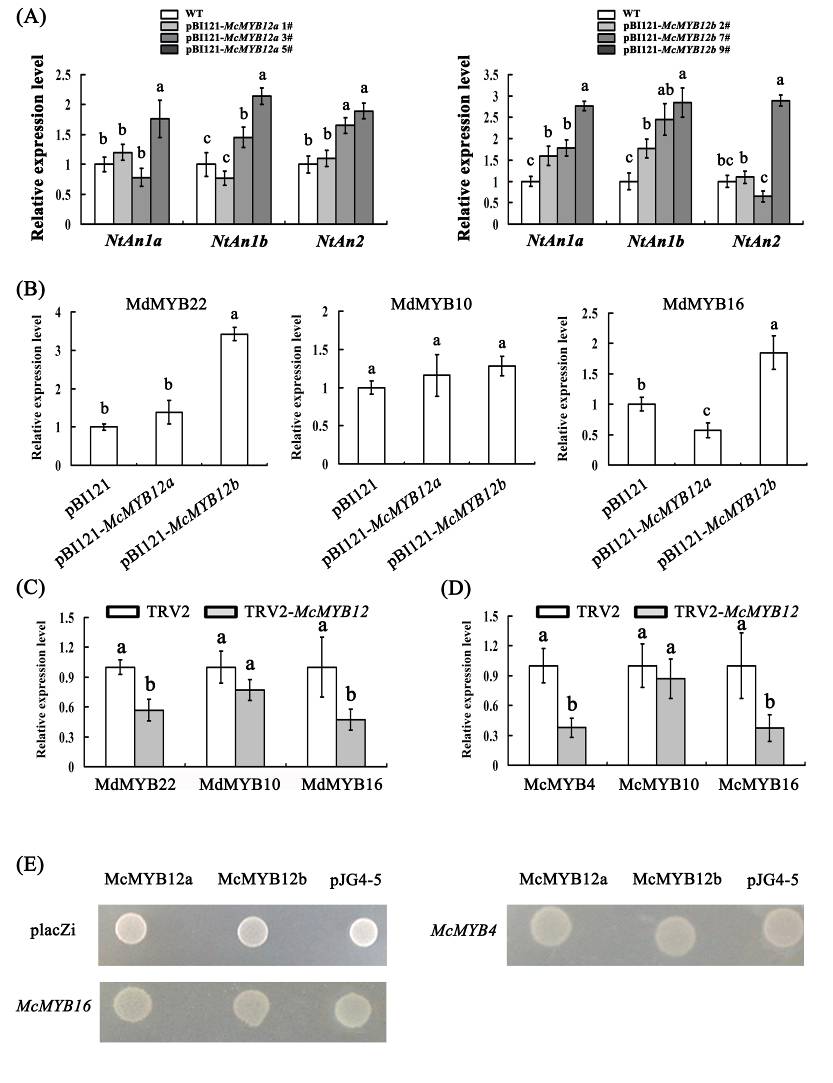


**Supplemental Figure 8. The relationship between McMYB12s with other MYB transcription factors.** (A) The expression level of *NtAn1a*, *NtAn1b* and *NtAn2* in leaves of *McMYB12s*- overexpressed tobacco leaves. Three different transgenic lines were analyzed. pBI121-*McMYB12a* 1#, pBI121-*McMYB12a* 3# and pBI121-*McMYB12a* 5#, three independent *McMYB12a*-overexpressing lines; pBI121-*McMYB12b* 2#, pBI121-*McMYB12b* 7# and pBI121-*McMYB12b* 9#, three independent *McMYB12b*-overexpressing lines. (B) The expression level of *MdMYB22*, *MdMYB10* and *MdMYB16* in *McMYB12s*- overexpressed apple peels. (C) The expression level of *MdMYB22*, *MdMYB10* and *MdMYB16* in *McMYB12s*- silenced apple peels. MdMYB22 have 99% amino acids sequence identity to McMYB4, MdMYB10 have 100% amino acids sequence identity to McMYB10 and MdMYB16 have amino acids 99% sequence identity to McMYB16. (D) The expression level of *McMYB4*, *McMYB10* and *McMYB16* in *McMYB12s*- silenced crabapple leaves. (E) Interaction of McMYB12 proteins with the promoters of McMYB4 and McMYB16.

**Supplemental Tables**

**Table S1** Primer sequences used in this study.

| **Accession**  **number** | **ID** | **Sequence (5’-3’)** | **Used for** |
| --- | --- | --- | --- |
|  |  |  |  |
| KJ020112 | pBI121-McMYB12a-F | GCTCTAGAATGAGGAACCCATCGTCTTCGT | pBI121-*McMYB12a* generation |
|  | pBI121-McMYB12a-R | GCTCTAGACTCCGTTTGATCATTAACGCTCTG |  |
| KJ020111 | pBI121-McMYB12b -F | ATCTAGAATGAGGAACCCGTCGTCTTC | pBI121-*McMYB12b* generation |
|  | pBI121-McMYB12b -R | ATCTAGACTGCTCCGTTTGATCAGTAACC |  |
|  | pTRV-McMYB12-F | TCTAGATGAGGAACCCATCGTCTTCG | TRV2-*McMYB12* generation |
|  | pTRV-McMYB12-R | GAGCTCTACTCCGTTTGATCATTAACGCTC |  |
| KY501104 | pMcMYB12a-F | GTCCTTTGCTGCTTCACTTCA | McMYB12a promoter cloning |
|  | pMcMYB12a-R | CGAATGCGAATACAAATGGAG |  |
| KY501103 | pMcMYB12b-F | AAGCCACTTACCTTTCGA | McMYB12b promoter cloning |
|  | pMcMYB12b-R | TTTGGTATTGAGGAGGATGG |  |
| KY501104 | G-McMYB12a-F | TCTCGAGTCCCACGCGC ATTCAA | pBI121-*proMcMYB12a*-GUS generation |
|  | G-McMYB12a-R | GCTCTAGACTGATCTTGT TTTTCTTACT TC |  |
| KY501103 | G-McMYB12b-F | ATCTCGAGGGCTATTAGC GTGCGT | pBI121-*proMcMYB12b*-GUS generation |
|  | G-McMYB12b-R | GCTCTAGAGGTATTGAGG AGGATGGGAG |  |
| KJ020112 | McMYB12a-F | CAGCAAGTGCTAAGATGCAAAC | qRT-PCR |
|  | McMYB12a-R | GCTATCAAAGACCACCGATTG |  |
| KJ020111 | McMYB12b-F | AGCAGCAAGTGCTACGATGAC | qRT-PCR |
|  | McMYB12b-R | GCTATCAAAGACCACCGATTG |  |
| FJ599763 | McCHS-F | TGACCGTCGAAGTTCGC | qRT-PCR |
|  | McCHS-R | TTTGTCACACATGCGCTGGA |  |
| FJ817485 | McCHI-F | AGGAGTTGTCGGAGTCCGTT | qRT-PCR |
|  | McCHI-R | ACTTTCTCAGAGTATTGCTGGCC |  |
| FJ817486 | McF3H-F | ACGAAGACGAGCGTCCAAAG | qRT-PCR |
|  | McF3H-R | CTCCTCCGATGGCAAAGCAA |  |
| KF481684 | McF3’H-F | CGTTGCTGTCGCTCACGGATGA | qRT-PCR |
|  | McF3’H-R | ATGACGTGTCAGTGCCAGCTGTG |  |
| FJ817487 | McDFR-F | CCGAGTCCGAATCCGTTTGT | qRT-PCR |
|  | McDFR-R | CCTTCTTCTGATTCGTGGGGT |  |
| FJ817488 | McANS-F | CACAGGGGCATGGTGAACAA | qRT-PCR |
|  | McANS-R | TTCACTTGGGGAGCAAAGCC |  |
| KF495603 | McUFGT-F | TGGGCGGACACCAATCA | qRT-PCR |
|  | McUFGT-R | ATGTCTCCACCGCACCA |  |
| KF495602 | McFLS-F | ACGAGCAACCGGGAATCACAACTG | qRT-PCR |
|  | McFLS-R | CCCAGTTGGAGCTGGCCTCAGTA |  |
| KT276930 | McANR1-F | AACCACAAGAAGGTCTCCCAC | qRT-PCR |
|  | McANR1-R | CCCTTGGATTGCTGGTTTGAT |  |
| KT276931 | McANR2-F | ACCCCTGTCAACTTTGCCTCA | qRT-PCR |
|  | McANR2-R | CCAAACCTGTTCCCTCAAGTGTAT |  |
| KT276929 | McLAR1-F | TTTATCAAAGGATGCCAGGTT | qRT-PCR |
|  | McLAR1-R | CATCCAAGGTCCTGAAAGAAT |  |
| KT276928 | McLAR2-F | TGGAGAAACGCAAGGTTAGAC | qRT-PCR |
|  | McLAR2-R | TCACCATAGATTTGGAACCGA |  |
| JX013493 | McMYB4-F | GACCAGCAGCAGAAACTA | qRT-PCR |
|  | McMYB4-R | ACAACCCTCCATTAATGCCGAC |  |
| JX162681 | McMYB10-F | ACGCCACCACAAACGTCGTCG | qRT-PCR |
|  | McMYB10-R | GGCGCATGATCTTGGCGACAGT |  |
| KP101181 | McMYB16-F | GCTCACACCAACAAAGGAGC | qRT-PCR |
|  | McMYB16-R | GCAGCTCTTCCCACATCGAA |  |
| DQ074470 | MdMYB22-F | GACCAGCAGCAGAAACTA | qRT-PCR |
|  | MdMYB22-R | ACAACCCTCCATTAATGCCGAC |  |
| ACQ45201 | MdMYB10-F | ACGCCACCACAAACGTCGTCG | qRT-PCR |
|  | MdMYB10-R | GGCGCATGATCTTGGCGACAGT |  |
| HM122617 | MdMYB16-F | GCTCACACCAACAAAGGAGC | qRT-PCR |
|  | MdMYB16-R | GCAGCTCTTCCCACATCGAA |  |
| DQ341382 | 18S RNA-F | GTCACTACCTCCCCGTGTCA | qRT-PCR |
|  | 18S RNA-R | GAGCCTGAGAAACGGCTACC |  |
| GQ339768 | NtActin-F | CGTTTGGACCTTGCTGG | qRT-PCR |
|  | NtActin-R | TCTGGGCAACGGAACCT |  |
| AF311783 | NtCHS-F | TCGGTCAAGCGGTTCAT | qRT-PCR |
|  | NtCHS-R | GTCATTGGGTCCACGAAAC |  |
| AB213651 | NtCHI-F | ATTGAAGGGAAGTTTGTGAAGT | qRT-PCR |
|  | NtCHI-R | ACCCGTCAAAGGCAAGA |  |
| AF036169 | NtF3H-F | CCAGATGGATGGATAGGTG | qRT-PCR |
|  | NtF3H-R | GGTAAGGTCGGGCTGTG |  |
| AB289449 | NtF3’H-F | CGTGATGGAATCCGACCTA | qRT-PCR |
|  | NtF3’H-R | AAGTCATTTCCTCGCACATC |  |
| AB289448 | NtDFR-F | GCCGCTGGTTGTTGGTC | qRT-PCR |
|  | NtDFR-R | CGCAGATGAATCTTCCCTC |  |
| AB723683 | NtANS-F | CTTTCTATTGGGCTGGGACT | qRT-PCR |
|  | NtANS-R | ACATCAGTATGAGCTTCAACGC |  |
| AB723686 | Nt3GT2-F | AGAAAGTTATGAAAGAGGCAGAG | qRT-PCR |
|  | Nt3GT2-R | GGACCGAATCAAATAAGTGTAT |  |
| AB289451 | NtFLS-F | GAACTTGAAGGGAAAAGGGG | qRT-PCR |
|  | NtFLS-R | TCCCTGTAGGAGGGAGGATT |  |
| AM827419 | NtLAR-F | TCAAGGTCCTTTACGCCATC | qRT-PCR |
|  | NtLAR-R | ACGAACCTGCTTCTCTTTGG |  |
| AM791704 | NtANR1-F | CATTTGACTTTCCCAAACGC | qRT-PCR |
|  | NtANR2-R | ATTGGGCTTTTGAGTTGTGC |  |
| DW003895 | NtANR2-F | TGTTCCCACTTGGGATGATA | qRT-PCR |
|  | NtANR2-R | TGCACCTATACTCTGTTAGTGGC |  |
| HQ589208 | NtAn1a-F | ACCATTCTCGAACACCGAAG | qRT-PCR |
|  | NtAn1a-R | TGCTAGGGCACAATGTGAAG |  |
| HQ589209 | NtAn1b-F | CTTGAACACTTCTCAAACCGA | qRT-PCR |
|  | NtAn1b-R | TGCTAGGGCACAATGTGAAG |  |
| FJ472647 | NtAN2-F | AGGAGAGCAAGCAAAAAGCA | qRT-PCR |
|  | NtAN2-R | TGAATTCCATCATCCGTCAA |  |
| CN944824 | MdCHS-F | GTGACTGTCCAGGAAGTTCGC | qRT-PCR |
|  | MdCHS-R | GCACACACTTGGATTCTCCTTTAG |  |
| CN946541 | MdCHI-F | GAAGGGTAAGACCGCCGAG | qRT-PCR |
|  | MdCHI-R | CACAATTCTCCGAAACTTTCTCAG |  |
| CN491664 | MdF3H-F | CGGGATGATGGGAAAACG | qRT-PCR |
|  | MdF3H-R | CGCTGGGTTCTGGAATGTG |  |
| CN491664 | MdF3’H-F | ACGATGGCGGATGTTACGG | qRT-PCR |
|  | MdF3’H-R | GCTTTGACCCTGCACTTGCT |  |
| AF117268 | MdDFR-F | GGACCCCGAGAATGAAGTG | qRT-PCR |
|  | MdDFR-R | CTCCACATTCACGGTTCCTG |  |
| AF117269 | MdANS-F | GAGAAGTATGCCAATGACCAGG | qRT-PCR |
|  | MdANS-R | GGCGGTTGCCTCAATGTAAT |  |
| AF119095 | MdFLS-F | ACGAGCAACCGGGAATCACAACTG | qRT-PCR |
|  | MdFLS-R | CCCAGTTGGAGCTGGCCTCAGTA |  |
| AF117267 | MdUFGT-F | GCTGACGAGTTGGGAGTGC | qRT-PCR |
|  | MdUFGT-R | CCTTCCGCTAAGTCTTTGATTC |  |
| DQ099803 | MdANR-F | GTTGCAACCCCTGTCAACTT | qRT-PCR |
|  | MdANR-R | CACGACCAAACCTGTTCCTT |  |
| DQ139836 | MdLAR-F | ACAACACCCACCCTTCTGAG | qRT-PCR |
|  | MdLAR-R | TGCAGCAAGGGCTAGTAGGT |  |
| GU396152 | pLacZi-*proMcCHS*-F | GGAATTCGGCGAGAATCAAACCTAA | pLacZi-*proMcCHS* generation |
|  | pLacZi-*proMcCHS*-R | CCCTCGAGGTGATACGAAAGTAATAGTCGG |  |
| KF481684 | pLacZi-*proMcF3’H*-F | CCGGAATTCTAGGTTTGGAGGCTCACGC | pLacZi-*proMcF3’H* generation |
|  | pLacZi-*proMcF3’H*-R | GGCCTCGAGTGGAGTTCAGTGTATAGTGGTGTTG |  |
| KT276932 | pLacZi-*proMcDFR*-F | GGAATTCATCTTGTGTGTATGTGCTTGCCGAA | pLacZi-*proMcDFR* generation |
|  | pLacZi-*proMcDFR*-R | CCGCTCGAGAGAAGTTTTAGGTTTGAATCTCGTGAATG |  |
| KT276926 | pLacZi-*proMcANS*-F | CCGGAATTCTGAAATCAGTCCTAAGAGTCAACGA | pLacZi-*proMcANS* generation |
|  | pLacZi-*proMcANS*-R | CCGCTCGAGATTTGGAGCTGGCTTTCGACAATAT |  |
| KT276927 | pLacZi-*proMcUFGT*-F | GGAATTCTGGATATCGGTGACCCTCC | pLacZi-*proMcUFGT* generation |
|  | pLacZi-*proMcUFGT*-R | CCTCGAGCTGAACTGGAGTGGACAGG |  |
| KT276925 | pLacZi-*proMcFLS*-F | GGAATTCTGCAGTTCAACGTCCTTAT | pLacZi-*proMcFLS*  generation |
|  | pLacZi-*proMcFLS*-R | CCCTCGAGAAAGAACGGTGGTGAACA |  |
| KT276921 | pLacZi-*proMcANR1*-F | CCGGAATTCTAAACAAAGGGCGAGAACCAA | pLacZi-*proMcANR1* generation |
|  | pLacZi-*proMcANR1*-R | CCGCTCGAGTGAAAAGGTAGGTGGGAATTATGGG |  |
| KT276922 | pLacZi-*proMcANR2*-F | GAATTCTCATAGTTAAACGAAGGGCGAGAACC | pLacZi-*proMcANR2* generation |
|  | pLacZi-*proMcANR2*-R | CTCGAGTGGCTTCCTCCTCCTCTGTCAGATT |  |
| KT276923 | pLacZi-*proMcLAR1*-F | CCGGAATTCTGCCCTGCGCAAACTCTCACAACTTG | pLacZi-*proMcLAR1* generation |
|  | pLacZi-*proMcLAR1*-R | CCGCTCGAGGGTAAACGAAAGCGCAAGGTTTCAC |  |
| KT276924 | pLacZi-*proMcLAR2*-F | CCGGAATTCAGAACAATCAACCGCAAGCAAT | pLacZi-*proMcLAR2* generation |
|  | pLacZi-*proMcLAR2*-R | CCGCTCGAGGTGATTTTTGACACGATTGTGGCAT |  |
| MDC001475.298 | pLacZi-*proMcMYB4*-F | AGGAATTCACCAATTTCGTGTTCCATTCGTC | pLacZi-*proMcMYB4* generation |
|  | pLacZi-*proMcMYB4*-R | ATCTCGAGGGCAACTTGTGCAAGTCAGA |  |
| EU518249 | pLacZi-*proMcMYB10*-F | AGGAATTCTGACCAAGTAAGCAGTGACCC | pLacZi-*proMcMYB10* generation |
|  | pLacZi-*proMcMYB10*-R | ATCTCGAG TTACAAGGCCAGTGACGTGC |  |
| MDC022732.149 | pLacZi-*proMcMYB16*-F | AGGAATTCCAGCTAGTGACATCTCTTCCCA | pLacZi-*proMcMYB16* generation |
|  | pLacZi-*proMcMYB16*-R | ATCTCGAGAGATGCGAGTTGGTGAGACAG |  |
| KJ020112 | pJG4-5-McMYB12a-F | CCGGAATTCATGAGGAACCCATCGTCTTCG | pJG4-5-*McMYB12a* generation |
|  | pJG4-5-McMYB12a-R | CCGCTCGAGCTACTCCGTTTGATCATTAACGC |  |
| GU396152 | pJG4-5-*McMYB12b*-F | CCGGAATTCATGAGGAACCCATCGTCTTCG | pJG4-5-*McMYB12b* generation |
|  | pJG4-5-*McMYB12b*-R | CCGCTCGAGCTACTGCTCCGTTTGATCAGTAAC |  |
| GU396152 | pGreen-*proMcCHS*-F | GCCATGGGGCGAGAATCAAACCTAA | pGreen-0800-*proMcCHS* generation |
|  | pGreen-*proMcCHS*-R | CCCCATGGGTGATACGAAAGTAATAGTCGG |  |
| KF481684 | pGreen-*proMcF3’H*-F | CCGCCATGGTAGGTTTGGAGGCTCACGC | pGreen-0800-*proMcF3’H*generation |
|  | pGreen-*proMcF3’H*-R | GGCCCATGGTGGAGTTCAGTGTATAGTGGTGTTG |  |
| KT276932 | pGreen-*proMcDFR*-F | GCCATGGATCTTGTGTGTATGTGCTTGCCGAA | pGreen-0800-*proMcDFR* generation |
|  | pGreen-*proMcDFR*-R | CCGCCATGGAGAAGTTTTAGGTTTGAATCTCGTGAATG |  |
| KT276926 | pGreen-*proMcANS*-F | CCGCCATGGTGAAATCAGTCCTAAGAGTCAACGA | pGreen-0800-*proMcANS* generation |
|  | pGreen-*proMcANS*-R | CCGCCATGGATTTGGAGCTGGCTTTCGACAATAT |  |
| KT276927 | pGreen-*proMcUFGT*-F | GCCATGGTGGATATCGGTGACCCTCC | pGreen-0800-*proMcUFGT* generation |
|  | pGreen-*proMcUFGT*-R | CCCATGGCTGAACTGGAGTGGACAGG |  |
| KT276925 | pGreen-*proMcFLS*-F | GCCATGGTGCAGTTCAACGTCCTTAT | pGreen-0800-*proMcFLS* generation |
|  | pGreen-*proMcFLS*-R | CCCCATGGAAAGAACGGTGGTGAACA |  |
| KT276921 | pGreen-*proMcANR1*-F | CCGCCATGGTAAACAAAGGGCGAGAACCAA | pGreen-0800-*proMcANR1*generation |
|  | pGreen-*proMcANR1*-R | CCGCCATGGTGAAAAGGTAGGTGGGAATTATGGG |  |
| KT276922 | pGreen-*proMcANR2*-F | ACCATGGTCATAGTTAAACGAAGGGCGAGAACC | pGreen-0800-*proMcANR2*generation |
|  | pGreen-*proMcANR2*-R | TCCATGGTGGCTTCCTCCTCCTCTGTCAGATT |  |
| KT276923 | pGreen-*proMcLAR1*-F | CCGCCATGGTGCCCTGCGCAAACTCTCACAACTTG | pGreen-0800-*proMcLAR1* generation |
|  | pGreen-*proMcLAR1*-R | CCGCCATGGGGTAAACGAAAGCGCAAGGTTTCAC |  |
| KT276924 | pGreen-*proMcLAR2*-F | CCGCCATGGAGAACAATCAACCGCAAGCAAT | pGreen-0800-*proMcLAR2*generation |
|  | pGreen-*proMcLAR2*-R | CCGCCATGGGTGATTTTTGACACGATTGTGGCAT |  |
| KJ020112 | pART7-*McMYB12a*-F | GCCTCGAGATGAGGAACCCATCGTCTTCGT | pART7-McMYB12a generation |
|  | pART7-McMYB12a-R | GCTCTAGACTCCGTTTGATCATTAACGCTCTG |  |
| KJ020111 | pART7-McMYB12b -F | ACTCGAGATGAGGAACCCGTCGTCTTC | pART7-McMYB12b generation |
|  | pART7-McMYB12b -R | ATCTAGACTGCTCCGTTTGATCAGTAACC |  |
| KJ020106 | pGreenII 62- SK- McbHLH3-F | ATGGATCCATGGCTGCACCGCCG | pGreenII 62- SK- McbHLH3 generation |
|  | pGreenII 62- SK- McbHLH3-R | GCGAATTCAGAGTCAGATTGGGGTA TAATTTG |  |
| KJ020112 | pSPY -McMYB12a-F | GCGGATCCATGAGGAACCCATCGTCTTCGT | BiFC assay |
|  | pSPY-McMYB12a-R | GCGGATCCCTCCGTTTGATCATTAACGCTCTG |  |
| KJ020111 | pSPY-McMYB12b -F | AGGATCCATGAGGAACCCGTCGTCTTC | BiFC assay |
|  | pSPY-McMYB12b -R | AGGATCCCTGCTCCGTTTGATCAGTAACC |  |
| HM122458 | pSPY-McbHLH3-F | CCGCGGATCCATGGCTGCACCGCCGCC | BiFC assay |
|  | pSPY-McbHLH3-R | CCGGGGTACCAGAGTCAGATTGGGGTAT |  |

**Table S2** Probe sequences used in this study.

| **Gene name** | **Probes (5’-3’)** | **Mutant Probes (5’-3’)** |
| --- | --- | --- |
| McCHS | GCAACTCCTCC**CAACTG**TGTGGATCAA  GCCACATACCCCGACTATTACT | GCAACTCCTCC**CATCAG**TGTGGATCAA  GCCACATACCCCGACTATTACT |
| McANS | AGATCATTGCTC**CAACTG**GCTAAAATT  AAGTGACCATTACTATAAT | AGATCATTGCTC**CTATTG**GCTAAAATT  AAGTGACCATTACTATAAT |
| McANR1 | ATTCTGAGTACTGT**AACCTAA**GTTGAA  GTTGAATAATTGACGTCGTC | ATTCTGAGTACTGT**AGCACAA**GTTGAAG  TTGAATAATTGACGTCGTC |
| McANR2 | ATTGGGAGATATGTGCTCGTTTTAGAG  TGCTTTTAAAA**TAACTG**AAAAC | ATTGGGAGATATGTGCTCGTTTTAGAGTG  CTTTTAAAA**TCAATT**AAAAC |
| McLAR1 | GGACAGAAAT**TATCC**ATACACCCTTTT  CGTTAATGTTGCAAACTGAGT | GGACAGAAAT**TGTAC**ATACACCCTTTTCG  TTAATGTTGCAAACTGAGT |
| McLAR2 | AAGTATGGTATAAACACAGACTAAGCC  TCATTATCA**TATCC**ATATCAT | AAGTATGGTATAAACACAGACTAAGCCTC  ATTATCA**GATAC**ATATCAT |

Core *cis*-element in the probes are shown in bold font.
